# An *IKBKE* variant conferring functional cGAS/STING pathway deficiency and susceptibility to recurrent HSV-2 meningitis

**DOI:** 10.1172/jci.insight.173066

**Published:** 2023-11-08

**Authors:** Azadeh Reyahi, Marie Studahl, Morten K. Skouboe, Stefanie Fruhwürth, Ryo Narita, Fanghui Ren, Moa Bjerhem Viklund, Marie B. Iversen, Mette Christiansen, Alexandra Svensson, Trine H. Mogensen, Kristina Eriksson, Søren R. Paludan

**Affiliations:** 1 Department of Rheumatology and Inflammation Research, Institute of Medicine, Sahlgrenska Academy, University of Gothenburg, Gothenburg, Sweden.; 2 Department of Biomedicine, Aarhus University, Aarhus, Denmark.; 3 Department of Infectious Diseases, Region Västra Götaland, Sahlgrenska University Hospital, Gothenburg, Sweden.; 4 Department of Infectious Diseases, Institute of Biomedicine, Sahlgrenska Academy, University of Gothenburg, Gothenburg, Sweden.; 5 Department of Molecular Medicine and; 6 Department of Infectious Diseases, Aarhus University Hospital, Aarhus, Denmark.

**Keywords:** Immunology, Infectious disease, Innate immunity

## Abstract

The mechanisms underlying susceptibility to recurrent herpes simplex virus type 2 (HSV-2) meningitis remain incompletely understood. In a patient experiencing multiple episodes of HSV-2 meningitis, we identified a monoallelic variant in the *IKBKE* gene, which encodes the IKKε kinase involved in induction of antiviral IFN genes. Patient cells displayed impaired induction of IFN-β1 (*IFNB1*) expression upon infection with HSV-2 or stimulation with double-stranded DNA (dsDNA) and failed to induce phosphorylation of STING, an activation marker of the DNA-sensing cyclic GMP-AMP synthase/stimulator of IFN genes (cGAS/STING) pathway. The patient allele encoded a truncated IKKε protein with loss of kinase activity and also capable of exerting dominant-negative activity. In stem cell–derived microglia, HSV-2–induced expression of *IFNB1* was dependent on *cGAS*, TANK binding kinase 1 (*TBK1*), and *IKBKE*, but not *TLR3*, and supernatants from HSV-2–treated microglia exerted *IKBKE*-dependent type I IFN–mediated antiviral activity upon neurons. Reintroducing wild-type *IKBKE* into patient cells rescued *IFNB1* induction following treatment with HSV-2 or dsDNA and restored antiviral activity. Collectively, we identify IKKε to be important for protection against HSV-2 meningitis and suggest a nonredundant role for the cGAS/STING pathway in human antiviral immunity.

## Graphical abstract

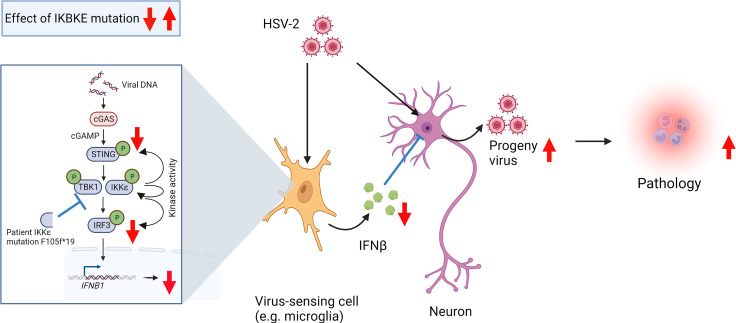

## Introduction

Herpes simplex virus type 2 (HSV-2) is a neurotropic virus of the alphaherpesvirus subfamily that usually manifests as genital herpes. The virus enters sensory nerve endings, reaches neuronal cell bodies, and establishes latent infection (1, 2). In rare cases, HSV-2 causes neurological disease represented by recurrent episodes of meningitis. Common symptoms include headache, fever, nausea, photophobia, and nuchal rigidity (3). In most cases, the symptoms resolve spontaneously within weeks, although some patients may develop recurrent neurological symptoms and neurocognitive impairments lasting for up to 1 year (4–7). In one-third of cases of primary HSV-2 meningitis, multiple recurrences may occur, leading to a highly disabling disease. Treatment with acyclovir or valaciclovir is a common therapy in the acute phase of disease and may be used in prevention of recurrent episodes (8). Although HSV-2 meningitis is a severe neurological disease associated with significant complications and reduced life quality, the pathophysiology of this condition is still largely unknown. Inborn errors of immunity have been suggested to underlie viral CNS infection in otherwise-healthy individuals (9), but precise knowledge of CNS disease determinants is yet to be completed.

Except for the protective role of HSV-1 antibodies against HSV-2 infection (10), little is known regarding the underlying predisposition to develop HSV-2 meningitis. However, multiple studies demonstrate genetically determined susceptibility to encephalitis caused by the other 2 members of the alphaherpesvirus subfamily, HSV-1 and varicella zoster virus (11–14). Therefore, it is believed that host genetic factors are central determinants for all alphaherpesviruses to disseminate into the CNS and cause disease. Identified genetic defects in individuals with herpes simplex encephalitis (HSE) and varicella zoster virus encephalitis include mutations in the sensing and signaling pathways for the pattern recognition receptors (PRRs) TLR3 and RNA polymerase III, leading to impaired type I IFN responses (11–21). These studies confirm a causal relationship between alphaherpesvirus encephalitis and defects in genes involved in type I IFN antiviral responses during both primary infection and reactivation from latency. Recently, inborn errors of immunity have been identified in individuals who develop HSV-2 meningitis, including rare host genetic variants in the autophagy machinery (22, 23). However, there are no data on HSV-2 meningitis and genes involved in PRR signaling, which raises the question of the role of the IFN system in control of HSV-2 infection, including reactivation from latency.

Type I IFNs are critical antiviral mediators induced by PRRs. Besides their contribution to elimination of viral infections, IFNs can cause pathological damage in the CNS (24–27). Type I IFNs are produced by many cell types in the CNS, with microglia being a major source (28). At the molecular level, PRR-mediated sensing of viral nucleic acids leads to activation of the kinase TANK binding kinase 1 (TBK1) and inhibitor of NF-κB kinase subunit epsilon (IKKε; gene name, *IKBKE*), 2 noncanonical members of IκB kinase (IKK) family, which in turn trigger phosphorylation of IFN regulatory factor 3 (IRF3). Eventually, this leads to nuclear translocation of IRF3 and transcription of IFNs and IFN-stimulated genes (ISGs) (29). The kinase activity of TBK1 and IKKε and activation of IRFs is tightly regulated to maintain immune system homeostasis. Structurally, TBK1 and IKKε have a similar domain composition including a kinase domain, a leucine zipper, a helix-loop-helix, and a ubiquitin-like domain. The proteins contain an ATP binding site and an active site, both of which are involved in the catalytic activity of the kinases (30–32). Herpesviruses are DNA viruses, and DNA is a potent stimulator of IFN responses, via sensing by cyclic GMP-AMP synthase (cGAS) and activation of signaling by the downstream adaptor protein stimulator of IFN genes (STING) (33, 34). A central step in activation of STING is the phosphorylation of S366 by TBK1 and IKKε, thus enabling recruitment of IRF3 to the adaptor protein for subsequent TBK1/IKKε-mediated phosphorylation and activation (35). Murine studies have suggested an important role for the cGAS/STING pathway in control of HSV-2 infections (36). However, the role of this immune pathway in antiviral defense in humans remains unresolved.

In this study, we present a genetic defect in a woman with recurrent HSV-2 meningitis by identification of a rare deleterious variant in *IKBKE*, which leads to functional defect of the cGAS/STING pathway, reduced phosphorylation of IRF3 and HSV-2–induced type I IFN production, and impaired control of virus replication in patient cells in agreement with the clinical phenotype.

## Results

### Clinical and demographic characteristics of patients with recurrent HSV-2 meningitis.

**Figure 1:**
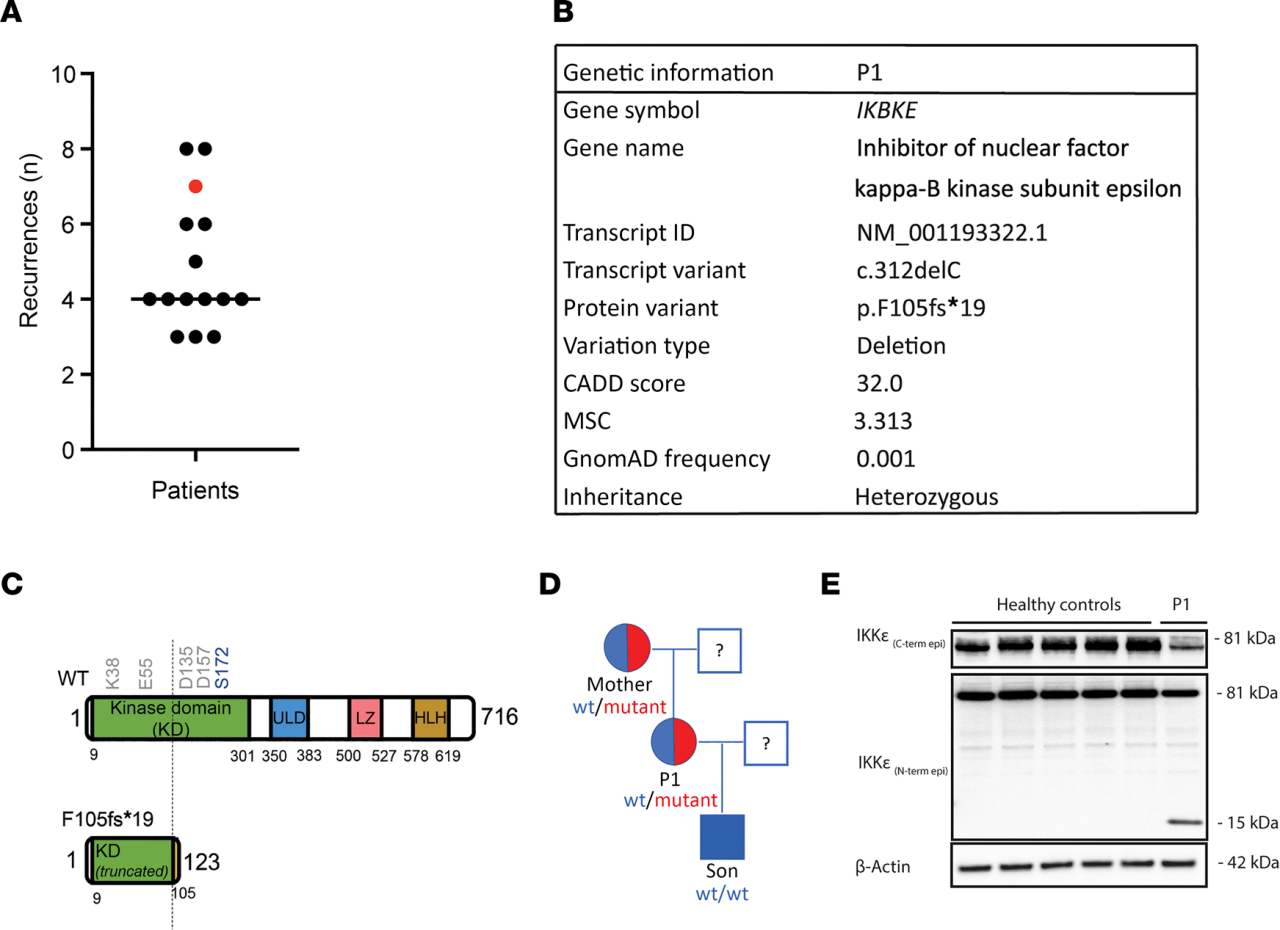
Identification of a monoallelic *IKBKE* variant in a patient with HSV-2 meningitis. (**A**) Number of disease episodes in the patients with recurrent HSV-2 meningitis. (**B**) Genetic information and characteristics of the *IKBKE* c.312delC variant identified in the patient. (**C**) Domain organization of WT IKKε and illustration of the localization of the mutation in the patient variant, including the resulting truncated F105f*19 protein. Key residues in the active site (shown in gray) and the phosphorylation target (shown in blue) are highlighted. ULD, ubiquitin-like domain; LZ, leucine zipper; HLH, helix-loop-helix. (**D**) Pedigree for the patient’s family revealed by Sanger sequencing. Family members heterozygous for the mutation are indicated by blue and red color. (**E**) Whole cell lysate of PBMCs from the patient and from healthy controls analyzed for IKKε protein level by immunoblotting using antibodies targeting epitopes in the N- and C-terminal parts of the protein.

We identified a patient (P1) in a cohort with the diagnosis of recurrent HSV-2 meningitis (Figure 1A). The patients in the cohort were diagnosed with HSV-2 meningitis based on clinical symptoms of viral meningitis and pleocytosis with lymphocytic predominance. HSV-2 DNA was detected in cerebrospinal fluid from the patient by PCR or showed positive HSV-2 serology together with virologically verified HSV-2 lesions in the genital or lumbosacral region (10, 37). Exclusion criteria were immunosuppression, such as HIV or treatment with immunosuppressive treatment. Recurrent HSV-2 meningitis was defined as at least 3 meningitis episodes fulfilling the criteria above. All recurrent episodes were confirmed by data from patients’ medical charts.

P1 was selected based on the genetic findings and the severe nature of the clinical story (Figure 1A). The patient is a 56-year-old female experiencing recurrent sacral HSV-2 infection with onset in her twenties. In her medical history, 7 episodes of HSV-2 meningitis have been documented. She was otherwise previously healthy with no history of recurrent or severe infections at the time of sampling. The most recent meningitis episode in 2018 led to a year-long rehabilitation period, and neuropsychology assessments showed cognitive deficits. Immunophenotyping of peripheral blood mononuclear cells (PBMCs) from P1 showed the profile to be largely within the normal range, except for minor alterations, including higher numbers of naive, recent thymic emigrants and Th1 cells (Supplemental Table 1; supplemental material available online with this article; https://doi.org/10.1172/jci.insight.173066DS1).

### Identification of a rare genetic variant in IKBKE gene.

Whole-exome sequencing (WES) was performed as previously described (22). Data were subsequently analyzed using key tools for predicting the deleterious nature of gene variants, including the Combined Annotation Dependent Depletion (CADD) score, gene damage index, and Mutation Significance Cutoff (MSC) (38, 39). The WES data analysis identified gene variants including both frameshift mutations (deletions) and nucleotide substitutions causing altered amino acid alterations. Additionally, the frequencies of variants were compared to publicly available databases (1000 Genomes, Genome Aggregation Database [gnomAD], and National Heart, Lung, and Blood Institute Exome Sequencing Project [NHLBI ESP]), and only variants with a frequency less than 0.01 were considered. Similar to the previously reported Danish cohort of recurrent HSV-2 meningitis (23), a STRING analysis grouped the variants identified in our cohort into biological pathways, including ubiquitin/proteasome pathways, autophagy machinery, and cell proliferation/apoptosis (Supplemental Figure 1A). Additionally, we identified a variant within genes associated with the antiviral type I IFN system. Particularly, we identified a monoallelic nucleotide deletion variant in *IKBKE* encoding IKKε in P1. Other variants were identified in this patient (Supplemental Table 2). These included a putative splice variant in DExH-box helicase 58 (*DHX58*) related to IFN expression, but we did not find altered expression levels of the *DHX58*-encoded LGP2 protein in patient cells compared to controls (Supplemental Figure 1B). The minor allele frequency of the *IKBKE* variant is less than 0.01, the CADD score is 32, and the gnomAD frequency is 0.001 (Figure 1B). The c.312delC variant (rs782190151) in *IKBKE* resulted in a frameshift in the amino acid sequence at position 105, which led to a premature stop codon and a truncated IKKε protein (designated F105f*19) consisting of only 123 residues rather than the 716 residues in the full-length WT protein. In the predicted structure of the truncated protein, the kinase domain, including the ATP binding site, is disrupted, as are key residues involved in enzymatic activity (Figure 1C) (40). The *IKBKE* c.312delC variant was confirmed by Sanger sequencing (Supplemental Figure 1C).

To decipher the pattern of inheritance and to determine whether the variant was generated de novo in P1, we isolated genetic material from the patient’s mother and son and sequenced *IKBKE*. The mother carries the same point mutation, whereas the son does not (Figure 1D). However, the mother is seronegative for HSV-2 infection and has not experienced meningitis. In addition, she has no medical history of severe complications from other viral infections. The serological status for infections with herpesviruses and some RNA viruses for P1, her mother, and her son is shown in Supplemental Table 3. P1 has antibodies against varicella zoster virus, morbilli, Epstein-Barr virus, and cytomegalovirus. Despite a record with a number of viral infections, HSV-2 meningitis is the only severe viral disease P1 has experienced.

To examine whether the *IKBKE* c.312delC variant was expressed, we performed immunoblotting analysis of lysates from patient PBMCs using antibodies directed against either the N- or C-terminal part of IKKε. We compared with 5 individual age- and sex-matched controls. We observed that WT IKKε protein was expressed at lower levels in P1 when probing with an antibody directed against the C-terminus of the IKKε protein (Figure 1E). A band of the predicted size of 15 kDa was found in patient PBMCs when probing with an antibody directed against the N-terminus of the IKKε protein, showing that the truncated IKKε F105f*19 protein is expressed (Figure 1E). In conclusion, we have identified an HSV-2 meningitis patient harboring a rare potentially disease-causing *IKBKE* variant giving rise to the expression of a truncated IKKε protein.

### Impaired IFNB1 response and STING activation following HSV-2 infection in PBMCs from P1.

**Figure 2:**
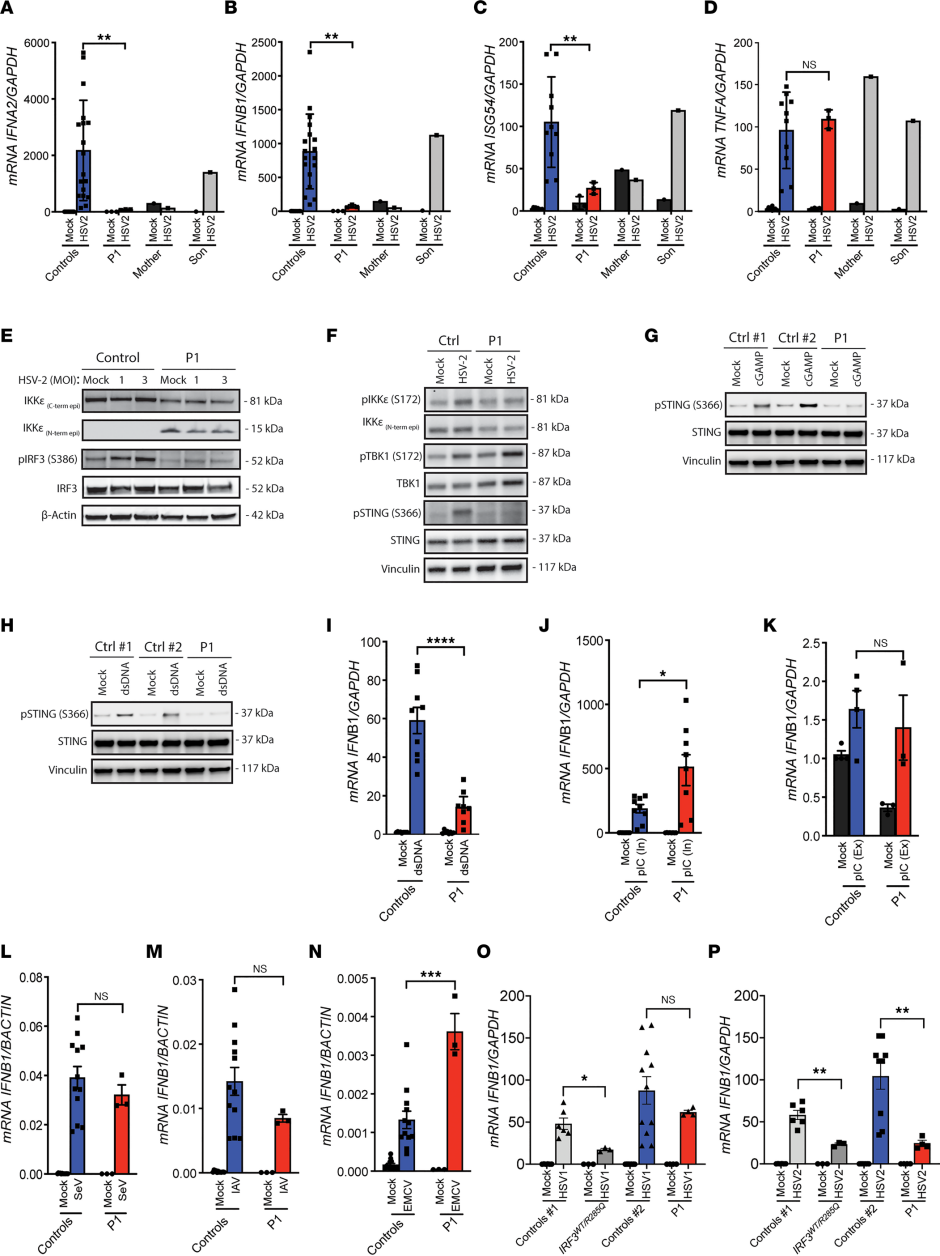
Impaired IFNB1 response and STING activation following HSV-2 infection in PBMCs from P1. (**A**–**D**) PBMCs were infected with HSV-2 with MOI 9. Total RNA was isolated after 6 hours for measurement of *IFNA2* (**A**), *IFNB1* (**B**), *ISG54* (**C**), and *TNFA* (**D**) mRNA by reverse transcription quantitative PCR (RT-qPCR). (**E** and **F**) Whole-cell lysates from PBMCs treated for 18 hours with HSV-2, as indicated, were examined for expression of IKKε (N- and C-terminal–targeting antibody), p-IKKε (S172), TBK1, p-TBK1 (S172), IRF3, p-IRF3 (S386), STING, and p-STING (S366). Data shown are representative of 4 (**E**) and 2 (**F**) independently performed experiments. (**G** and **H**) Fibroblasts were transfected with cGAMP or dsDNA (4 μg/mL) for 4 hours, and lysates were immunoblotted for p-STING (S366), STING, and Vinculin. (**I**–**K**) PBMCs were stimulated with dsDNA (4 μg/mL) or poly(IC) (4 μg/mL intracellular, 50 μg/mL extracellular). (**L**–**P**) PBMCs were infected for 6 hours with Sendai virus (SeV), influenza A virus (IAV) (MOI 0.1), encephalomyocarditis virus (EMCV) (MOI 0.1), HSV-1 (MOI 3), and HSV-2 (MOI 3), and *IFNB1* mRNA was quantified by qPCR. All measurements were done in triplicates, relative to housekeeping genes *GAPDH* or *BACTIN* and normalized to the pooled uninfected PBMCs from 10 (**A**–**D**), 9 (**I**, **J**, and **L**–**N**), 4 (**K**), 2 young (control 1), and 4 around 50 years old (control 2) (**O** and **P**) healthy controls. Results were obtained from 3 (**A**–**D**) (except for mother and son PBMCs, which were examined once) and (**I**–**N**) or 2 (**O** and **P**) independent experiments. The nonparametric Mann-Whitney (**A**–**D**) and unpaired *t* test (**I**–**P**) were used to evaluate statistical significance between groups. Error bars represent standard error of mean (SEM). *, *P* ≤ 0.05; **, *P* ≤ 0.01; ***, *P* ≤ 0.001; ****, *P* ≤0.0001.

To start examining the functional impact of the identified *IKBKE* c.312delC variant on the innate immune response to HSV-2, PBMC from P1, her mother, her son, and age- and sex-matched healthy controls were infected with HSV-2 and evaluated for transcription of the genes *IFNA2*, *IFNB1*, *ISG54*, and *TNFA*. PBMCs from P1 expressed lower levels of *IFNA2, IFNB1,* and *ISG54* mRNA in response to HSV-2 infection, although the levels of *TNFA* mRNA were comparable between infected patient and control cells (Figure 2, A–D, and Supplemental Figure 2, A–D). A similar pattern was observed in PBMCs from the patient’s mother, but not in PBMCs from her son, potentially suggesting an association between harboring of the *IKBKE* mutation and impaired antiviral type I IFN responses. Since IKKε plays a role in the IFN-inducible antiviral transcriptional response through phosphorylation of IRF3 (41), we next examined whole-cell protein lysates from infected PBMCs for virus-induced phosphorylation of IRF3. Patient PBMCs failed to phosphorylate IRF3 in response to HSV-2 infection (Figure 2E and Supplemental Figure 2, E–H). A broader characterization of the signaling response in HSV-2–infected PBMCs showed that the patient cells did not induce phosphorylation of IKKε at residue S172, required for kinase activation, or of STING at residue S366, which is required for recruitment of IRF3 and induction of *IFNB1* expression (Figure 2F and Supplemental Figure 2, I–M). The finding of reduced levels of phosphorylated STING prompted us to explore whether cells from P1 have defective activation of STING in response to synthetic activators of STING. Indeed, P1 cells exhibited impaired phosphorylation of S366 after stimulation with cyclic GMP-AMP or dsDNA (Figure 2, G and H). In agreement with this, patient PBMCs showed low *IFNB1* induction following dsDNA treatment (Figure 2I). Surprisingly, P1 PBMCs showed no impairment of the response to the dsRNA mimic poly(IC) in comparison to healthy controls (Figure 2, J and K) and were also able to mount *IFNB1* induction in response to a panel of RNA viruses (Figure 2, L–N).

HSV-1 infection of P1 PBMCs led to *IFNB1* mRNA expression statistically indistinguishable from the response in control PBMCs (Figure 2O), potentially suggesting a central role for TLR3 in sensing of HSV-1 in this cell type. We have previously reported a heterozygous loss-of-function mutation in IRF3 in a patient with HSE (18). When comparing HSV-1– and HSV-2–induced *IFNB1* induction in PBMCs from P1 and the patient with the IRF3 mutation, we found that cells from the patient with IRF3 deficiency showed impaired *IFNB1* induction by both alphaherpesviruses (Figure 2, O and P). Collectively, cells harboring the *IKBKE* c.312delC variant have an impaired ability to activate signaling via IKKε, STING, and IRF3 following HSV-2 infection and DNA stimulation, thus impeding the expression of *IFNB1* and ISGs.

### Impaired IFNB1 expression and increased viral load in HSV-2–infected P1 fibroblasts.

**Figure 3:**
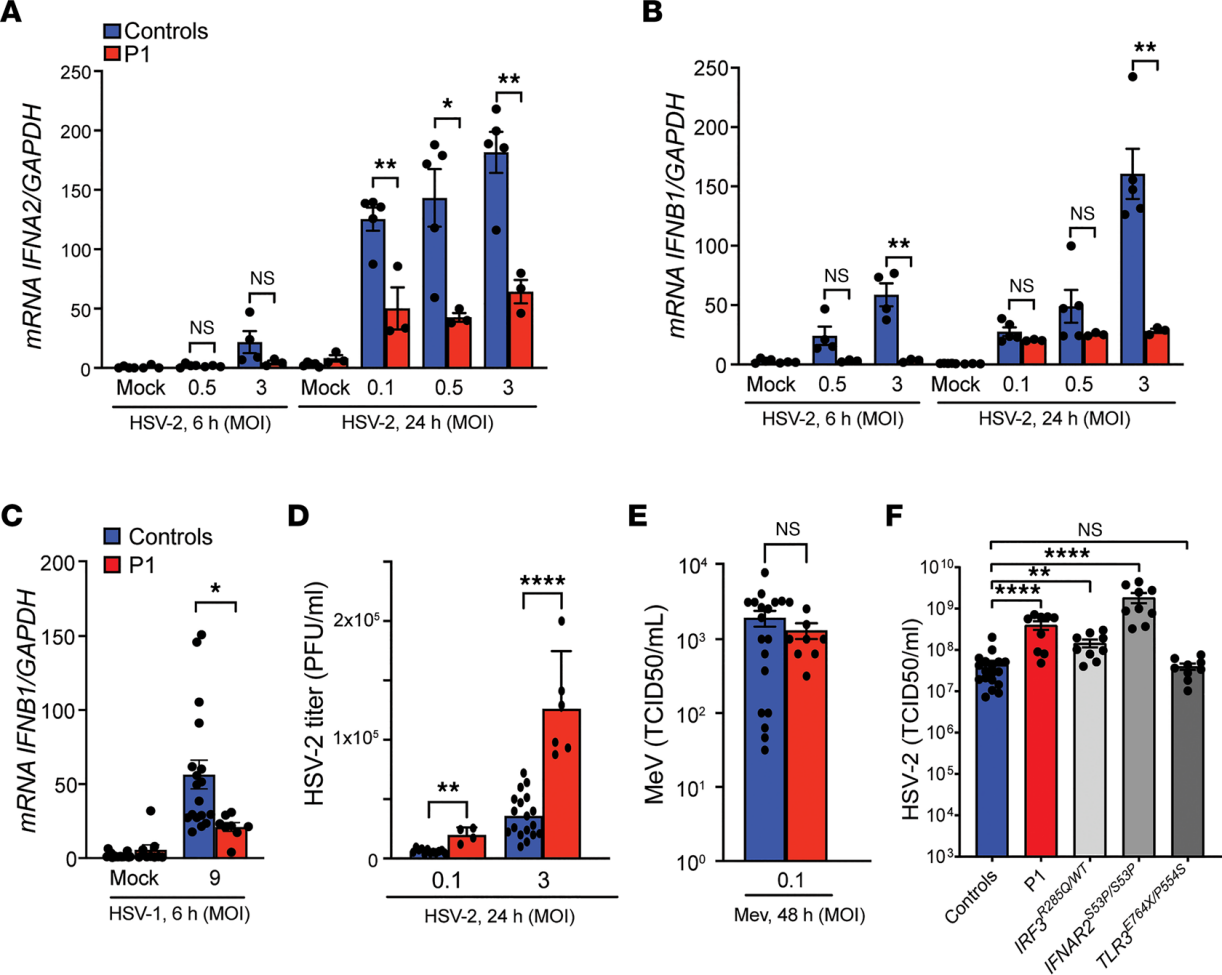
Impaired IFNB1 expression and increased viral load in HSV-2–infected P1 fibroblasts. (**A** and **B**) Fibroblasts were infected with HSV-2. Total RNA was harvested after 6 and 24 hours and subjected to RT-qPCR for measurement of *IFNA2* (**A**) and *IFNB1* (**B**) mRNA. Cytokine mRNA levels were normalized to *GAPDH*, and the patient was compared with either 4 controls (for 6-hour stimulation) or 5 controls (for 24-hour infection). Unpaired *t* test was used for statistical analysis. (**C**) Fibroblasts were infected with HSV-1. Total RNA was harvested after 6 hours for *IFNB1* mRNA quantification. (**D**) A plaque assay was used to measure HSV-2 titers in supernatants after 24 hours of infection. Nonparametric Mann-Whitney rank-sum test was used for statistical analysis. (**E**) Fibroblasts from P1 and 2 healthy controls were infected with measles virus (MeV) for 48 hours. A TCID_50_ (50% tissue culture infectious dose) assay was used to determine MeV titers in supernatants. Unpaired *t* test was used for statistical analysis. (**F**) Fibroblasts from P1 and patients functionally deficient in IRF3, IFNAR2, or TLR3 and fibroblasts from 2 healthy controls were infected with HSV-2 (MOI 1) for 24 hours, and a TCID_50_ assay was performed to quantify viral titers. Data were analyzed with 1-way ANOVA, and Dunnett’s multiple comparisons test was used for statistical analysis. Data shown in **A** and **B** are representative of 3 independent experiments, while the data in **C** represent 1 of 2 independent experiments. Data shown in **D**–**F** are pooled from 3 independent experiments. Error bars representing SEM and *, *P* ≤ 0.05; **, *P* ≤ 0.01; ****, *P* ≤ 0.0001.

To address whether a broader range of patient cell types had impaired host innate immune responses and reduced ability to control HSV-2 replication, fibroblasts were isolated from patient skin biopsies and infected with HSV-2. Similar to what was observed in PBMCs, patient fibroblasts showed impaired virus-induced expression of *IFNA2* and *IFNB1* mRNA at both early and late time points postinfection (Figure 3, A and B). The P1 fibroblasts also evoked reduced *IFNB1* expression to HSV-1 infection, which was not observed in PBMCs (Figure 3C). In agreement with the reduced *IFNB1* expression, plaque assays on supernatants showed significantly enhanced HSV-2 load in cultures from P1 fibroblasts compared with controls (Figure 3D and Supplemental Figure 3A). This was not observed after infection with measles virus, which harbors its genome in the form of RNA (Figure 3E). Importantly, fibroblasts from IRF3-deficient and interferon α and β receptor subunit 2–deficient (IFNAR2-deficient) patients, but not from a TLR3-deficient patient (18, 42, 43), supported elevated HSV-2 replication (Figure 3F). Treatment of P1 fibroblasts with recombinant IFN-β reduced viral replication with the same efficiency as in control fibroblasts (Supplemental Figure 3B). These results demonstrate defective anti–HSV-2 defense in P1 fibroblasts and link defective IFNB1 expression with increased susceptibility to HSV-2 replication.

### The IKKε F105f*19 protein is unable to stimulate IFN induction.

**Figure 4:**
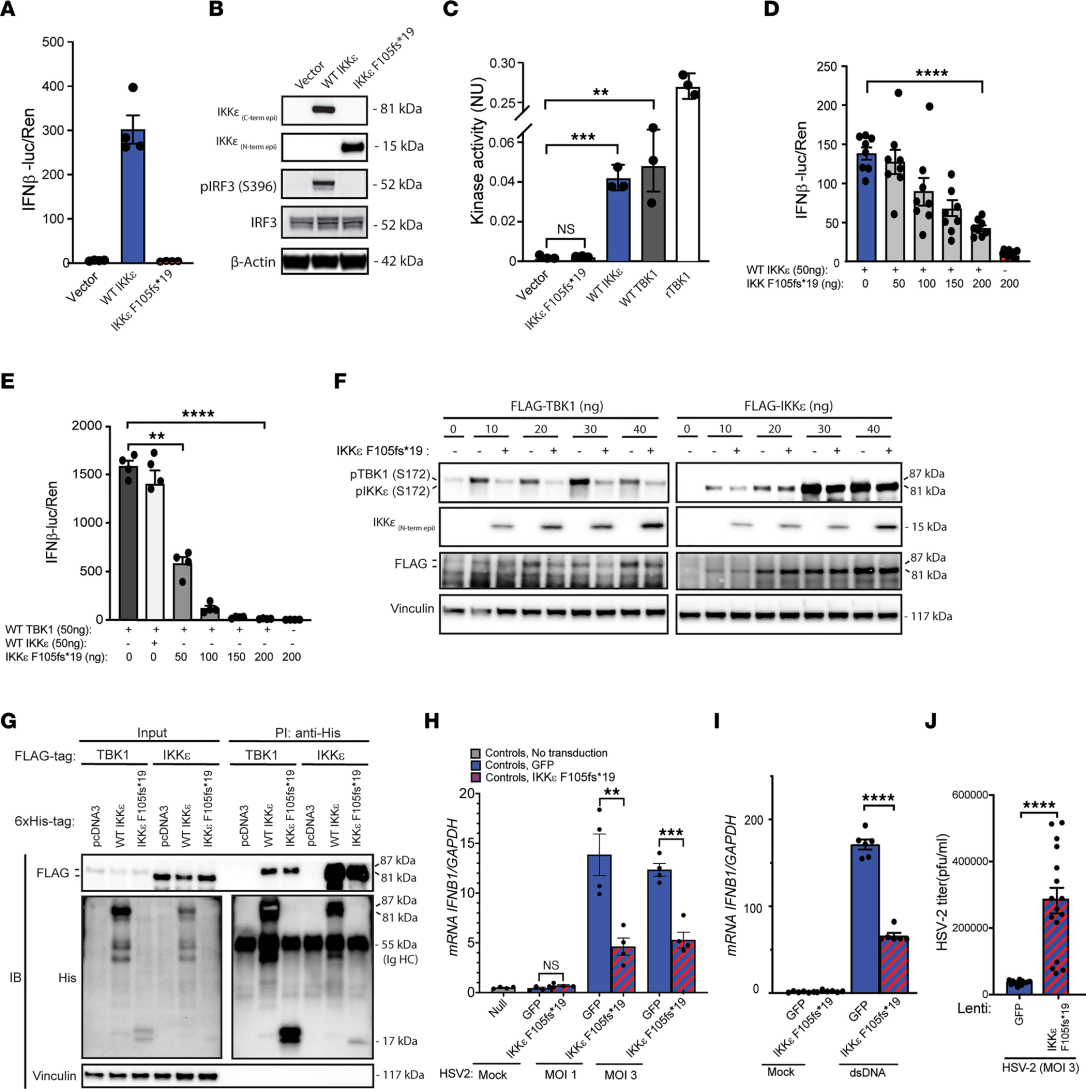
The IKKε F105f*19 protein is unable to stimulate IFN induction. (**A**) HEK293T cells were transiently transfected with IFN-β promoter firefly, β-actin promoter renilla reporter plasmids, and 100 ng of vector and plasmids encoding the full-length (WT) and truncated P1 F105fs*19 IKKε to measure IFN-β gene expression indicated by luciferase activity after 16 hours. (**B**) p-IRF3 (S396) and total IRF3 in whole-cell lysates from HEK293T cells expressing empty vector, WT, and P1 variant IKKε. IKKε _(N/C-term epi)_, antibodies targeting epitopes in the N- and C-terminal parts of IKKε. (**C**) Lysates from HEK293T cells transfected with the indicated expression plasmids were immunoprecipitated with anti-FLAG and subjected to assays determining IKKε kinase activity. Data are presented as normalized levels of luciferase signals from the ATP measurements. (**D** and **E**) HEK293T cells were transfected with IFN-β promoter firefly, β-actin promoter renilla reporter plasmids, WT IKBKE, WT TBK1, and the indicated amounts of P1 IKBKE. (**F**) HEK293T cells were transfected with P1 IKBKE, and the indicated amounts of plasmids encoding FLAG-TBK1 and FLAG-IKKε. Cells were lysed 24 hours later and immunoblotted for p-TBK1 (S172), p-IKKε (S172), FLAG, IKKε (probing of only low molecular range of blotted membrane), and Vinculin. (**G**) HEK293T cells were transfected with 6x-His-WT or P1 IKBKE, FLAG-TBK1, and FLAG-IKBKE. Lysates were immunoprecipitated with anti-His and immunoblotted with anti-FLAG. (**H**–**J**) Control fibroblasts were transduced with lentiviral vectors encoding patient IKKε variant F105fs*19 (MOI: 12), infected with HSV-2 at the indicated MOI for 6 hours (**H**) or 24 hours (**J**), or transfected with dsDNA (**I**) for 6 hours. Data presented are from 1 representative of 6 (**A**, **D**, and **E**), 2 (**F** and **G**), and 3 (**B**, **C**, and **H**–**J**) independently performed experiments. Nonparametric Mann-Whitney rank-sum test was used for statistical analysis. Error bars represent SEM. **, *P* ≤ 0.01; ***, *P* ≤ 0.001; ****, *P* ≤ 0.0001. NU = (Luc_max_ – Luc_measured_)/Luc_max_ (see Methods).

To investigate the functional impact of the protein product F105fs*19 of the identified P1 *IKBKE* c.312delC variant, we expressed cDNA encoding WT and the P1 IKKε variant in HEK293T cells and confirmed by immunoblotting that both forms were expressed and with the predicted sizes (Supplemental Figure 4A). Next, we expressed the proteins together with a firefly IFN-β luciferase gene and a β-actin renilla reporter as internal control. Overexpression of WT IKKε led to activation of the *IFNB1* promoter, whereas the P1 IKKε F105fs*19 variant had no effect on/did not induce the *IFNB1* promoter activity (Figure 4A). Consistent with these results, phosphorylation of IRF3 was induced in lysates from HEK293T cells overexpressing WT IKKε but not in lysates from cells overexpressing the P1 IKKε variant (Figure 4B), and the IKKε F105fs*19 protein had no kinase activity (Figure 4C). To examine whether the patient IKKε F105fs*19 variant exhibited dominant-negative activity, we cotransfected a constant amount of WT *IKBKE* plasmids together with increasing amounts of plasmids with the P1 *IKBKE* c.312delC variant. The WT IKKε–induced activation of the *IFNB1* promoter was significantly decreased with increasing transfection of the *IKBKE* c.312delC variant, suggesting the P1 IKKε F105f*19 protein expressed from the patient allele possesses dominant-negative activity (Figure 4D). IKKε has identical domain organization and structure as TBK1, and the 2 kinases have high sequence homology (Supplemental Figure 4B). The 2 kinases display functional overlap in the induction of type I IFN expression. We also examined the impact of P1 IKKε F105f*19 protein on TBK1-activated IFNB1 transcription. To this end, we cotransfected HEK293T cells with *TBK1* and increasing amounts of the P1 *IKBKE* c.312delC variant and observed inhibition of the TBK1-induced IFNB1 reporter activity even more potently than the response induced by transfection of *IKBKE* (Figure 4E). Consistent with this, expression of IKKε F105f*19 potently blocked accumulation of TBK1 phosphorylated on S172 and only more modestly blocked phosphorylation of IKKε on S172 (Figure 4F). However, we also observed that in the HEK293T cell system, the levels of P1 IKKε variant F105f*19 were much lower when coexpressed with WT IKKε compared with when coexpressed with WT TBK1 (Supplemental Figure 4C). This complicates the interpretation of the reporter gene data with respect to relative dominant-negative effect of P1 IKKε F105f*19 protein on TBK1 versus IKKε. Importantly, and despite the low expression of the P1 IKKε F105f*19 protein when coexpressed together with WT IKKε, we observed that both TBK1-FLAG and IKKε-FLAG were co-immunoprecipitated with His-tagged F105f*19 (Figure 4G).

To examine whether the P1 IKKε F105f*19 protein exerted dominant-negative activity in primary cells, we transduced control fibroblasts with the P1 *IKBKE* c.312delC variant. First, flow cytometry and fluorescence microscopy showed a high transduction efficiency (Supplemental Figure 4, D and E). Importantly, transduction of *IKBKE* c.312delC into control fibroblasts reduced their ability to express *IFNB1* in response to HSV-2 and dsDNA and also impaired control of HSV-2 replication (Figure 4, H–J). Corroborating this, expression of the P1 IKKε F105f*19 protein in control fibroblasts reduced HSV-2–induced accumulation of phosphorylated STING, while this was augmented upon overexpression of WT IKKε (Supplemental Figure 4F). Collectively, these data suggest that the *IKBKE* c.312delC mutation in P1 leads to functional deficiency of the resulting protein product IKKε F105f*19 and contribution from dominant-negative activity against WT IKKε and TBK1.

### IKBKE, TBK1, and cGAS are important for IFNB1 induction in microglia after HSV-2 exposure.

**Figure 5:**
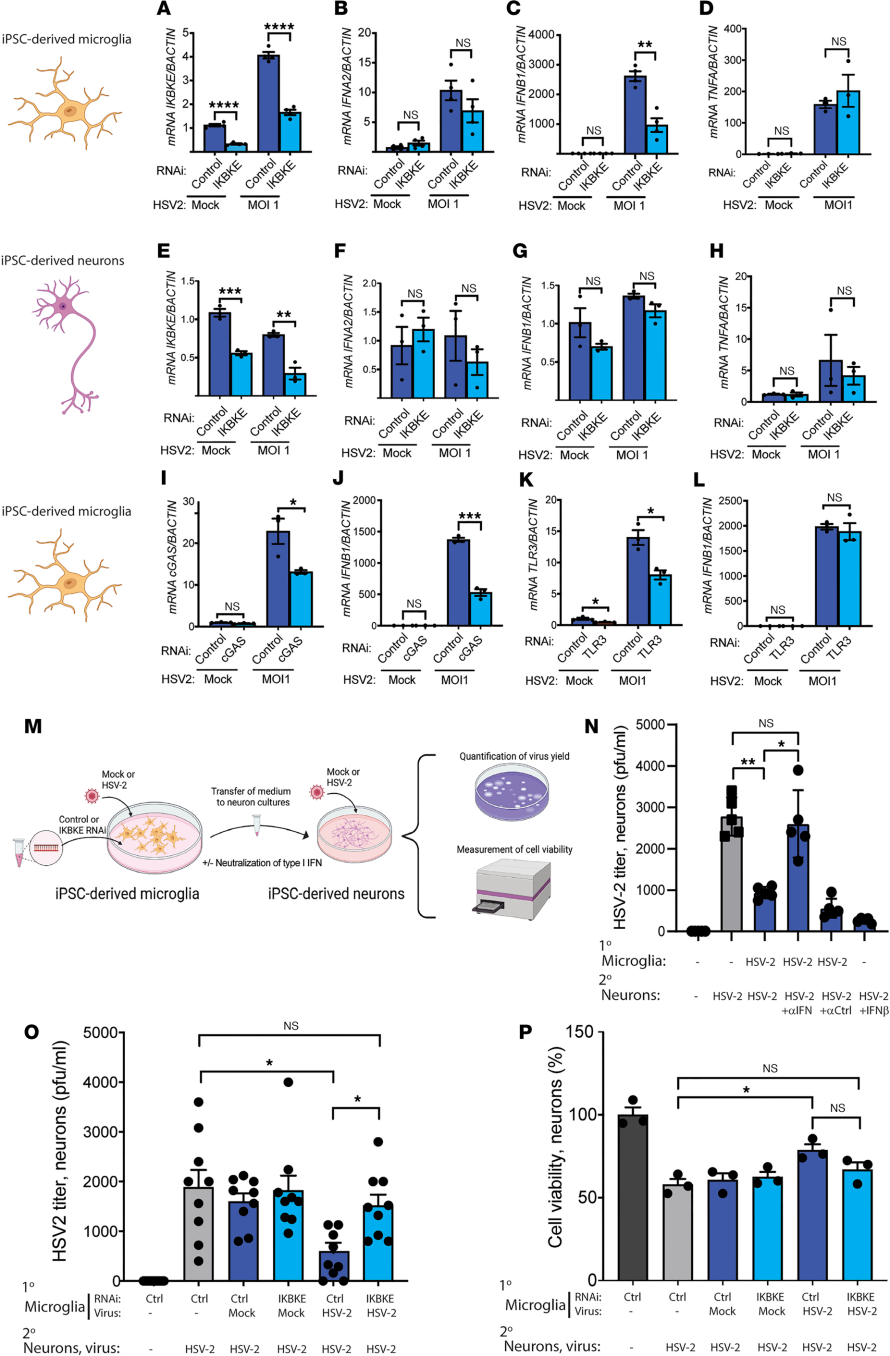
IKBKE, TBK1, and cGAS are important for IFNB1 induction in microglia after HSV-2 exposure. (**A**–**H**) iPSC derived microglia (**A**–**D**) and neurons (**E**–**H**) were transfected with *IKBKE* or control siRNA. (**A** and **E**) The efficiency of transfection was examined by measuring *IKBKE* mRNA levels. Cells were infected with HSV-2 for 6 hours, and *IFNA2*, *IFNB1*, and *TNFA* mRNA levels were quantified. (**I**–**L**) iPSC-derived microglia were transfected with siRNA targeting cGAS (**I** and **J**) or TLR3 (**K** and **L**). Microglia were infected with HSV-2, and *IFNB1* mRNA was quantified (**J** and **L**). Unpaired *t* test was used for statistical analysis. (**M**) Graphical illustration of experimental setup for microglia-neuron crosstalk experiments. Created with BioRender.com. (**N**) iPSC-derived neurons were treated with Human Type 1 IFN Neutralizing Antibody Mixture or control IgG (both 1:100) 30 minutes before addition of supernatants from HSV-2–infected microglia or treatment with IFN-β (10 ng/mL). Six hours later, the medium was removed, and the neurons were infected with HSV-2. Supernatants were collected after 16 hours for plaque assay (**O**). Microglia subjected to *IKBKE* or control knockdown with siRNA were infected with HSV-2. The cells were washed, and the medium was replaced after 1 hour. Supernatants were collected from the microglia after 24 hours and added to the neurons. Six hours later, the medium was removed, and the neurons were infected with HSV-2. Supernatants were collected 16 hours later for plaque assay. Data presented are pooled from 2 independently performed experiments. (**P**) Cells were treated as in panel **N**, and culture supernatants were analyzed for cell viability. Data presented are from 1 of 2 experiments performed. (**N**–**P**) Groups were compared with Brown-Forsythe and Welch ANOVA with Dunnett’s T3 multiple comparisons test. Error bars represent SEM (**A**–**H**) and SD (**N**–**P**), and *, *P* ≤ 0.05; **, *P* ≤ 0.01; ***, *P* ≤ 0.001; ****, *P* ≤ 0.0001.

Microglia represent a major contributor to the type I IFN response in the CNS (28). We therefore examined whether reduced IKKε expression in microglia and in neurons, the HSV-2 target cells, would affect innate antiviral responses. To this end, we differentiated microglia and neurons from human induced pluripotent stem cells (iPSCs) (Supplemental Figure 5, A and B). Targeting of *IKBKE* expression by siRNA in iPSC-derived microglia led to significantly reduced *IKBKE* expression, with about 75% reduction in mock-infected cells and about 60% reduction in HSV-2–infected cells (Figure 5A). HSV-2 infection of iPSC-derived microglia induced the expression of both *IFNA2* and *IFNB1* (Figure 5, B and C). *IKBKE* depletion in these iPSC-derived microglia was associated with a reduced HSV-2–induced expression of *IFNB1* but not *IFNA2* and *TNFA* (Figure 5, B–D). In contrast, we did not observe any HSV-2–induced expression of *IFNA2*, *IFNB1*, or *TNFA* in iPSC-derived neurons and consequently no effect on *IKBKE* knockdown on type I IFN expression in these cells (Figure 5, E–H). Since we observed a dominant-negative effect of the P1 variant on TBK1 activity in HEK293T cells, we also examined the effect of TBK1 knockdown and small molecule–based inhibition of TBK1/IKKε on HSV-2–induced gene expression in microglia. Targeting of TBK1 expression reduced HSV-2–induced expression of both *IFNB1* and *TNFA*, thus suggesting overlapping as well as distinct functions of these 2 kinases in activation of immune responses to HSV-2 infection in microglia (Supplemental Figure 5, C–E). As expected, inhibition of both kinases largely abrogated HSV-2–induced *IFNB1* expression in microglia (Supplemental Figure 5, F and G). Next, we targeted cGAS and TLR3, 2 proposed sensors of HSV-2 (36, 44), in order to evaluate which PRRs detect HSV-2 infection and signal through IKKε/TBK1. Interestingly, we observed that reduction of cGAS expression, but not TLR3 expression, significantly impaired HSV-2–induced IFNB expression in microglia (Figure 5, I–L). In support of this, inhibition of cGAS with a small molecule inhibitor blocked HSV-2–induced *IFNB1* expression (Supplemental Figure 5H). Thus, human microglia sense HSV-2 through cGAS to exert IKKε/TBK1-dependent signaling and *IFNB1* expression. Although siRNA and small molecule inhibitors do not fully reflect the proposed mechanism of action of the P1 IKKε F105f*19 variant, particularly not the dominant-negative activity, these data support the results from P1 cells that reduced IKKε activity leads to reduced virus-induced IFNB1 expression.

To examine the impact of IKKε on antiviral microglia-neuronal crosstalk, we next isolated culture media from uninfected and HSV-2–infected control and *IKBKE*-knockdown iPSC-derived microglia to prevent HSV-2 infection of iPSC-derived neurons (Figure 5M). The supernatants from the HSV-2–exposed iPSC-derived IKKε WT microglia contained less than 0.1% of the infectious dose used to infect the iPSC-derived neurons in vitro. Pretreatment of neurons with supernatants from HSV-2–infected microglia led to reduced viral yield in the supernatants, and this was blocked by treatment with neutralizing type I IFN antibody mixture (Figure 5N). Importantly, the reduction in viral yield in neurons was not observed when they were treated with supernatants from microglia with *IKBKE* knockdown (Figure 5O). The HSV-2–infected neurons receiving supernatants from microglia cultures were partially protected from cell death, and this was dependent on full *IKBKE* expression in the microglia (Figure 5P). These data suggest that IKKε-dependent type I IFN responses in microglia can exert paracrine protection against HSV-2 infection in neurons and prevent infection-induced neuronal cell death.

### Overexpression of WT IKKε in the patient’s fibroblasts restores the antiviral immune response.

**Figure 6:**
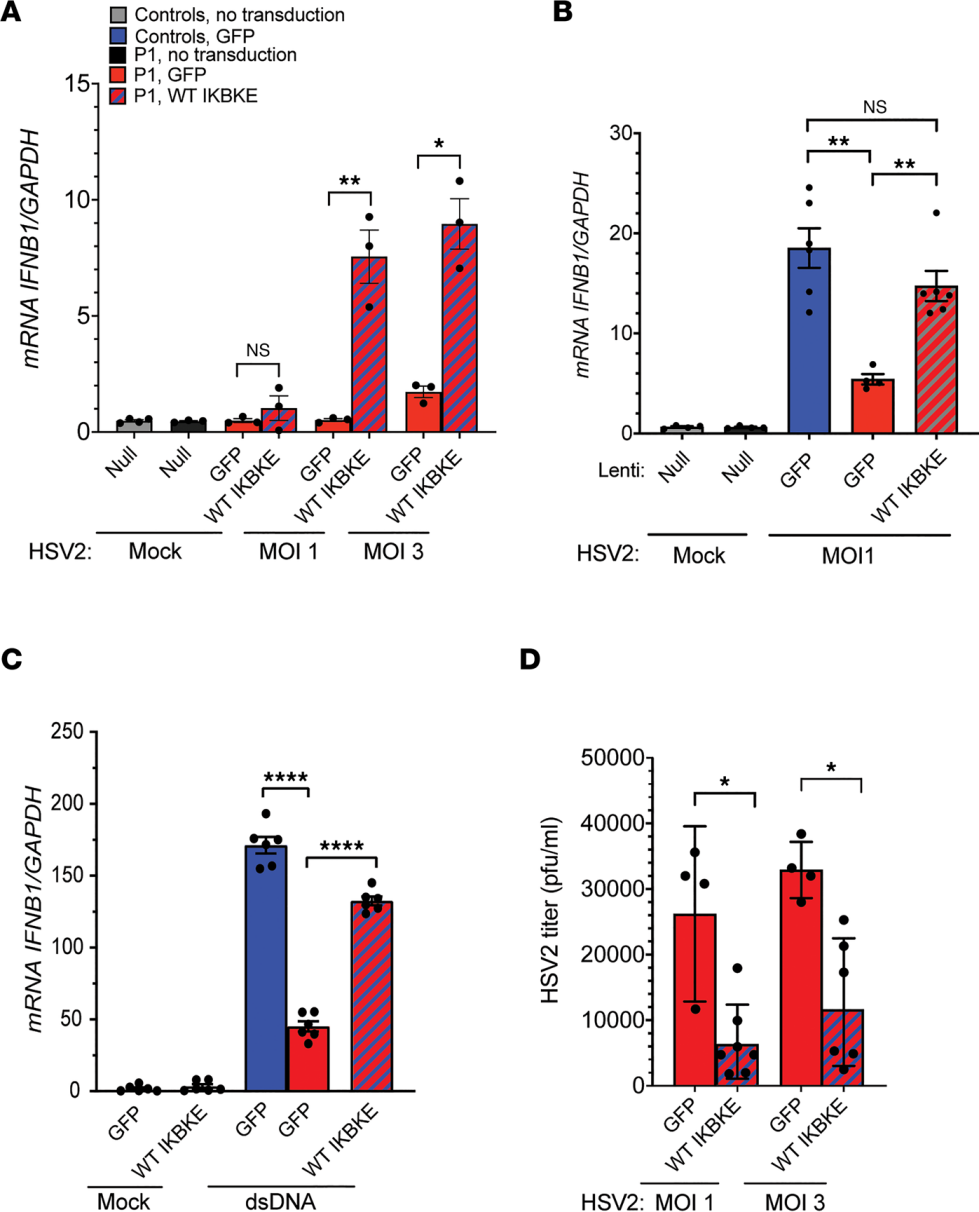
Reconstitution of WT *IKBKE* in patient fibroblasts rescues anti–HSV-2 response. Fibroblasts from P1 and controls were transduced with lentiviral vectors encoding WT IKKε or GFP (MOI of 12). (**A** and **B**) Transduced fibroblasts were infected with HSV-2 at MOI of 1 and 3 for 6 hours or (**C**) were transfected with dsDNA (4 μg/mL) for 6 hours. Total RNA was harvested from the cell lysates and subjected to RT-qPCR for measurement of *IFNB1* mRNA level. Data presented are from 1 representative of 3 independent experiments performed. (**D**) Supernatants were collected from the cells 24 hours after infection for quantification of viral load by plaque assay. Data presented are merged of 2 independent experiments performed. (**A** and **D**) Unpaired *t* test was used for statistical analysis. (**B** and **C**) Groups were compared with Brown-Forsythe and Welch’s ANOVA with Dunnett’s T3 multiple comparisons test. Error bars represent SD and *, *P* ≤ 0.05; **, *P* ≤ 0.01; ****, *P* ≤ 0.0001.

To examine whether reintroduction of WT *IKBKE* into primary fibroblasts from P1 restored HSV-2–induced IFNB1 responses and control of HSV-2 infection, we transduced P1 fibroblasts with lentiviral vectors encoding either GFP (control vector) or WT. In some instances F105fs*19 IKKε was also included as already shown in Figure 4. We first confirmed the expression of IKKε proteins following the lentiviral transduction in fibroblasts and observed that the levels of WT IKKε protein reached levels comparable to control fibroblasts after transduction (Supplemental Figure 6A). Importantly, transduction of P1 fibroblasts with WT *IKBKE* rescued their ability to produce *IFNB1* in response to HSV-2 exposure (Figure 6A) and to a level comparable to GFP-transduced control cells (Figure 6B). This treatment also restored the ability of P1 cells to induce full IFNB1 expression in response to treatment with the cGAS/STING pathway agonist dsDNA (Figure 6C). In agreement with the restored IFNB1 response to HSV-2 infection in P1 cells upon transduction with the WT *IKBKE* allele, this led to significantly lower levels of HSV-2 replication in these cells (Figure 6D). As expected, transduction of P1 cells with *IKBKE* c.312delC did not reconstitute virus-induced IFNB1 expression or control of virus replication (Supplemental Figure 6, B and C). Collectively, these data establish a causal relationship between the *IKBKE* c.312delC mutation, reduced type I IFN responses, and impaired control of HSV-2 replication.

## Discussion

We identified an adult HSV-2 meningitis patient with an autosomal-dominant frameshift loss-of-function mutation in *IKBKE* leading to reduced virus-induced induction of type I IFN-stimulated genes and impaired control of HSV-2 replication. Importantly, siRNA knockdown in iPSC-derived microglia demonstrated that HSV-2 induced antiviral responses in a manner dependent on cGAS, IKBKE, and TBK1, and cells from the patient were unable to activate cGAS/STING signaling in response to synthetic DNA or HSV-2 infection. Since the patient has experienced at least 7 recurrences of HSV-2 meningitis, these data suggest an important role for IKKε and the cGAS/STING pathway in control of HSV-2 CNS reactivation and CNS invasion. This is, to the best of our knowledge, the first report of a human infectious disease linked to an IKBKE variant.

Recognition of pathogen-associated molecular patterns by host PRRs results in activation of innate immune signaling pathways. Upon sensing of viral DNA and RNA, e.g., by cGAS and TLR3, signaling is initiated, which leads to activation of TBK1 and IKKε. These 2 kinases have a similar domain composition and share 67% amino acid identity (45, 46). In contrast to TBK1, which is widely expressed in almost all cell types, IKKε is more selectively expressed and is inducible, being upregulated by, e.g., cytokines (40, 47, 48). *IKBKE*-deficient mice are viable, although they are hypersusceptible to some viral infections (41, 49), whereas *TBK1*-knockout mice die at embryonic day 15 from TNF-induced apoptosis in the liver (50). Since TBK1 is an essential component in the type I IFN signaling pathway, it would be expected that the loss of TBK1 would lead to an immunocompromised state in humans. Indeed, Herman et al. reported heterozygous loss-of-function mutations in 2 individuals with HSE (19). The patient with HSV-2 meningitis we report here carries a frameshift variant in *IKBKE* leading to a premature stop codon within the kinase domain of the molecule and expression of a truncated nonfunctional protein with dominant-negative effect on IKKε and TBK1 activity. IKKε and TBK1 are dimeric proteins in the active state, and the dimer is formed through multiple interactions involving residues in the truncated kinase domain contained in the P1 IKKε F105fs*19 variant (51, 52). Indeed, we found that the P1 IKKε F105fs*19 variant could immunoprecipitate WT IKKε and TBK1, and reduce kinase activity, as well as activation of *IFNB1* reporter gene expression in HEK293T cells. These data argue in favor of formation of inactive dimers with IKKε and TBK1 being the mode of action of the P1 IKKε variant. The degree to which the P1 IKKε F105fs*19 protein exerts dominant-negative activity on both WT IKKε and TBK1 in primary cells was not fully resolved in this work, but we noted that phosphorylation of IKKε, but not TBK1, was reduced in P1 PBMCs exposed to HSV-2. Although the breadth of the dominant-negative activity of the P1 IKKε F105fs*19 protein across cell types was not fully resolved in this study, we did find that expression of the protein in skin fibroblasts from healthy donors strongly impaired HSV-2–induced *IFNB1* expression and control of virus replication. Based on these data, we conclude that the IKKε F105fs*19 protein is a loss-of-function variant and that dominant-negative activity of this protein likely also contributes to the immunological phenotype in the patient cells.

There are several genetic studies showing single-gene inborn errors of the TLR3 or IFN pathway in adults or children with HSE (14–19), which is in line with the idea that both tonic and inducible levels of IFN-β in the CNS, and thereby ISGs in cortical neurons, protect the CNS from HSE during primary infection (53). One recent study now also suggests a role for the TLR3/IRF7 pathway in recurrent HSE in infants (54). With respect to HSV-2, previous studies have shown an important role for different PRRs and type I IFN signaling in control of this virus in mouse models of acute infection (36, 44). In this study, we observed a significant cellular phenotype in patient cells with HSV-2 exposure evoking reduced activation of STING signaling and *IFNB1* expression and impaired antiviral activity. This was not observed following HSV-1 infection, suggesting the mutation does not confer susceptibility to HSE, consistent with the mother being HSV-1 positive and not having a disease history. We also observed that patient cells had reduced responsiveness to synthetic DNA, which was restored upon reconstitution with WT *IKBKE* and that targeting of *cGAS*, *IKBKE*, or *TBK1* expression with RNAi in microglia reduced HSV-2–induced *IFNB1* expression. The phosphorylation of STING at S366 is mediated by dimeric TBK1/IKKε and enables recruitment of IRF3 for subsequent phosphorylation by the same kinases and transcriptional activation of the IFN response (35). Our data suggest that the genetic alteration in P1 impairs the ability of the cGAS/STING pathway to respond to HSV-2 genomic DNA with activation of IKKε, and possibly TBK1, thus impeding activation of the IRF3-driven antiviral response. The observed selectivity of the P1 IKKε F105fs*19 protein for inhibiting the cGAS/STING pathway remains to be understood in detail.

Another interesting observation from this study is that neurons do not evoke IFN responses upon HSV-2 infection and hence rely on paracrine activity to induce IFN-mediated antiviral protection. Indeed, supernatants from HSV-2–infected microglia exerted antiviral activity on neurons, and this could be neutralized with neutralizing type I IFN antibodies. The HSV-2–induced type I IFN response in microglia was significantly reduced upon *IKBKE* knockdown. Hence, loss of full IKKε function disrupts the paracrine antiviral IFN system between, e.g., microglia and neurons, hence leading to increased viral replication in neurons, possibly reflected in increased susceptibility to viral meningitis in the patient.

The patient had antibodies against several common RNA and DNA viruses. Since we observed unaltered *IFNB1* response to synthetic RNA and RNA viruses, RNA-sensing pathways appear to be largely intact, and it is therefore not surprising that the patient has not developed severe disease upon infection with RNA viruses. It is also worth noting that P1 had largely unaltered composition of blood cells, which combined with the detectable levels of HSV-2 antibodies, may contribute to explaining why the repeated HSV-2 reactivations did not lead to systemic infections. The observation that the patient is seropositive for a large range of herpesviruses without having a history of severe infections is interesting and leads to a series of findings. First, there is significant redundancy in the innate immunological pathways controlling DNA viruses. Second, different herpesviruses may exhibit different sensitivity to IFNs and defects in innate immunity. Third, innate immunological pathways may be involved in controlling latency of some, but not other, herpesvirus infections. This work shows how an inborn error of immunity in a type I IFN induction pathway predisposes to recurrent meningitis caused by HSV-2. This information may be relevant when developing therapies for interferonopathies, by targeting the cGAS/STING pathway and the IFN system.

It is important to note that the cellular model systems used in this study have limitations. HSV-2 meningitis is a disease caused mainly by viral reactivation, whereas the in vitro model systems used here resemble acute infection. Despite this limitation, we do believe that the proposed effect of the *IKBKE* c.312delC variant on type I IFN responses applies broadly across most or all cell types, since we observed loss of IFN induction in (i) patient cells, (ii) iPSC-derived microglia targeted with *IKBKE* siRNA, (iii) control fibroblasts transduced with the *IKBKE* c.312delC variant, and (iv) HEK293T cells transfected with the *IKBKE* c.312delC variant. However, the detailed cellular/molecular mechanisms explaining why the *IKBKE* c.312delC variant resulted in recurrent HSV-2 meningitis and no other severe manifestations of viral infections remain elusive. Finally, we cannot distinguish whether meningitis developed because the patient had a higher viral latent reservoir due to impaired control of primary infection, impaired control of viral latency through reduced type I IFN response by, e.g., satellite glial cells, or compromised innate immune control of viral spread in the meninges following reactivation.

Recently, Hait et al. characterized rare monoallelic variants in the autophagy proteins ATG4A and LC3B2 in 2 patients with recurrent HSV-2 meningitis and suggested an important role of autophagy in anti–HSV-2 immunity. The weak IFN response to HSV-2 infection in iPSC-derived neurons supports the hypothesis that neurons require other immune mechanisms, e.g., autophagy, to exert antiviral activity (55, 56). Nevertheless, our data highlight the importance of type I IFN responses in protection against HSV-2 in the CNS, including a nonredundant role in the prevention of disease evoked during viral reactivation. Collectively, this finding provides genetic and immunologic evidence for the role of IKKε, cGAS/STING signaling, and type I IFN in antiviral immunity to HSV-2 in the CNS.

## Methods

### Study design.

This study included a cohort of 48 patients (39 women, 9 men; ≥18 years of age) with recurrent HSV-2 meningitis. Genomic DNA from 15 patients with recurrent HSV-2 meningitis was subjected to WES. The mean age of these patients was 49.5 years (range 39–58), and there were 12 women and 3 men. They had on average 4.9 (median 4) episodes of meningitis previously (range 3–8) (Figure 1A). Seven patients were treated with acyclovir or valaciclovir prophylaxis against recurrent HSV-2 infection (genital/sacral, or meningeal) at the time of sampling. The patient described here (P1) is a 56-year-old woman of Caucasian (Swedish) origin. All patients in the cohort were diagnosed with HSV-2 meningitis and recruited at the Department of Infectious Diseases, Sahlgrenska University Hospital in Sweden between April 2013 and October 2015.

### Clinical case summary.

P1 was identified based on the clinical history of more than 3 episodes of recurrent viral meningitis confirmed by pleocytosis in cerebrospinal fluid (levels of > 5 × 10 ^6^ cells/L) and preceding or concurrent virologically verified HSV-2 lesions in the genital or lumbosacral region. P1 was selected due to the identified rare gene variant involved in antiviral defense and the high number of recurrences. She was otherwise previously healthy at the inclusion with no history of other recurrent or severe infections. At the age of 23, she had her first meningitis episode. Lumbar puncture showed mononuclear pleocytosis. She had frequent HSV-2 sacral blisters, virologically verified by virus isolation from blisters, and IgG antibodies against HSV-2 in serum, and was diagnosed with HSV-2 meningoencephalitis. In periods she had several recurrences of genital and/or sacral herpes lesions and was treated with oral acyclovir. The patient experienced a long period of postmeningitis symptoms, mainly cognitive deficits as well as photophobia, after the fourth episode of meningitis. Electroencephalogram showed low-frequency activity over the anterior quadrant of the left hemisphere with dominance on the anterior temporal lobe, suggestive of meningoencephalitis, and low-frequency activity in the temporal lobe in the right hemisphere. MRI of the brain revealed a 5 mm cyst in the left putamen. MRIs were performed at regular intervals, showing that the volume of the cyst was unchanged and single minimal punctate bleedings in the left temporal lobe and medially in the right occipital lobe had appeared. The most recent meningitis episode in 2018 was followed by a period of rehabilitation, and neuropsychology assessments showed cognitive deficits. The following years she was again on antiviral prophylaxis with acyclovir, which was subsequently switched to valaciclovir.

### WES and bioinformatics.

WES was conducted on genomic DNA isolated from whole blood from each patient in the cohort. WES was performed using the KAPA HTP Library Preparation and Nimblegen SeqCap EZ MedExome Plus kits, then further analyzed with the Illumina NextSeq 550 system. Single nucleotide polymorphisms were called relative to hg19 with Burrows-Wheeler Aligner. Variants were analyzed by with Ingenuity Variant Analysis (QIAGEN) and filtered as described previously (57).

### Isolation, culture, and stimulation of primary cells.

Blood samples from P1, her mother, and her son were collected both for DNA extraction and subsequent WES and/or Sanger sequencing and for isolation of PBMCs for immunological evaluations. As controls, we used blood from 10 age- and sex-matched healthy Swedish adults. PBMCs were purified from heparin-stabilized blood by a Ficoll density gradient centrifugation and stored in FBS (Invitrogen) containing 15% DMSO and frozen at –150°C until use. Frozen PBMCs were thawed in DMEM (Gibco) containing 10% FBS, 1% l-glutamine (G7513, MilliporeSigma), and 1% Penicillin/Streptomycin (P0781, MilliporeSigma) and incubated for 8 hours at 37°C before stimulations. For stimulations, PBMCs were treated with dsDNA (4 μg/mL, LCG Biosearch Technologies), 2′3′-cGAMP (2 μg/mL, InvivoGen, tlrl-nacga23m), or poly(IC) (4 μg/mL intracellular, 50 μg/mL extracellular, InvivoGen, tlrl-picw) using Lipofectamine 2000 (Life Technologies, 11668-030). Primary dermal fibroblasts were obtained from 4 mm skin biopsies, cultured, and expanded in complete DMEM. Fibroblasts from patients deficient in *IRF3*, *IFNAR2*, and *TLR3* were described previously (18, 42, 43). Recombinant IFN-β was obtained from PeproTech and used as the indicated concentrations. For inhibition of TBK1 and IKKε or cGAS in iPSC-derived microglia, cells were pre- and cotreated with Amlexanox (150 μg/mL, InvivoGen inh-amx), BX795 (10 μM, InvivoGen, trlr-bx7), or RU.521 (2 μg/mL, InvivoGen inh-ru521) from 2 hours before the viral infection.

### Viruses.

For in vitro experiments in PBMCs and fibroblasts, viral infections were performed with HSV-2 (333 strain), HSV-1 (McKrae strain), measles virus (Edmonston strain, ATCC, VR-24), Sendai virus (Cantrell strain), influenza A virus (clinical isolate of Hamburg 2009/H1N1), and encephalomyocarditis virus (EMC Florida strain) in different concentrations and time points as indicated in each experiment. The viruses were propagated on permissive cells and titrated as described previously (12, 42).

### RNA purification and reverse transcription quantitative PCR.

Total RNA was isolated from cells using RNase Mini kit (74106, QIAGEN), followed by cDNA synthesis using High-Capacity cDNA Reverse Transcription Kit (4368814, Applied Biosystems) following the manufacturer’s instructions. TaqMan probes (Life Technologies) were used to measure the mRNA expression levels of the following genes: *IKBKE* (Hs01063858), *IFNA2* (Hs00265051), *IFNB1* (Hs01077958), *ISG54* (Hs01922738), *TBK1* (Hs00179410), *cGAS* (Hs00403553), *TLR3* (Hs01551079), *TNFA* (Hs01113624), *Bactin* (Hs1060665), and *GAPDH* (Hs02758991).

### Immunoblotting and antibodies.

To quantify the protein expression levels, cells were lysed on ice using RIPA buffer completed with DTT and phosphatase and protease inhibitor (Roche). Supernatant containing proteins was collected after 10 minutes’ centrifugation at 13,000*g* at 4°C. Protein was mixed with 4× sample buffer (NP0007, Invitrogen), heated for 10 minutes at 70°C, loaded on a 4%–20% acrylamide gel (NW04120BOX), and transferred to a nitrocellulose membrane (IB23002, Invitrogen). The membrane was blocked in 5% BSA in TBS-Tween 20 (TBST) buffer for 1 hour on a shaker at room temperature and then incubated with primary antibody overnight on a shaker at 4°C. Following washing, the membrane was incubated with HRP-conjugated secondary antibody for 1 hour at room temperature, developed with Clarity Western ECL substrate (170-5060, Bio-Rad), and visualized by ChemiDoc gel imaging system (Bio-Rad). The following primary and secondary antibodies were used: rabbit anti-IKKε (C-terminus) (Cell Signaling Technology, D20G4), mouse anti-IKKε (N-terminus) (Santa Cruz Biotechnology, sc-376114), IRF3 (Cell Signaling Technology, 11904), LGP2 (Invitrogen, PA5-20433), p-IRF3 S396 (Cell Signaling Technology, 4947), TBK1 (Cell Signaling Technology, 3504S), p-TBK1 S172 (Cell Signaling Technology, 5483), STING (Cell Signaling Technology, 13647S), p-STING S366 (Cell Signaling Technology, 19781S), p-IRF3 S386 (Cell Signaling Technology, 37829S), p-IKKε S172 (Cell Signaling Technology, 8766S), NF-κB p65 (Cell Signaling Technology, 8242S), p–NF-κB p65 (Cell Signaling Technology, 3033), IκBα (Cell Signaling Technology, 9242S), LC3B (Cell Signaling Technology, 2775S), CL-CASP3 (Cell Signaling Technology, 9661S), His (Cell Signaling Technology, 12698S), FLAG (MilliporeSigma, F1804), HRP-conjugated β-actin antibody (MA5-15739-HRP, Invitrogen), GAPDH (Cell Signaling Technology, 5175), and Vinculin (MilliporeSigma, V9131). The secondary antibodies were purchased from Jackson ImmunoResearch: peroxidase-conjugated F(ab′)_2_ donkey anti-mouse IgG (H+L) (1:10,000), peroxidase-conjugated F(ab′)_2_ donkey anti-rabbit IgG (H+L) (1:10,000), and HRP-conjugated goat anti-rabbit and rabbit anti-mouse secondary antibodies (Dako).

### Kinase assay.

HEK293T cells (ATCC) were transfected with plasmids encoding Flag-tagged WT IKKε, WT TBK1, F105f*19 IKKε, or empty vector. Cells were lysed 24 hours posttransfection and immunoprecipitated with anti-Flag precipitate from 1 × 10^6^ cells and were analyzed for kinase activity using the Kinase-Glo Max Assay (Promega, V6071). As positive control recombinant TBK1 (PBS Biosciences, 40286) was used. The assay was incubated for 60 minutes. Luminescence was recorded on a Synergy LX Multi-Mode Microplate Reader. Data are presented as (Luc_max_ – Luc_measured_)/Luc_max_ and called NUs.

### Luciferase reporter assays.

HEK293T cells were seeded in 96-well plates at a density of 65,000 cells/well. Then, 24 hours later, the cells were transiently transfected with 45 ng/well of firefly luciferase reporter plasmid (under the control of the IFN-β promoter), 5 ng/well of renilla luciferase reporter plasmid (under the control of the β-actin promoter), and different concentrations of plasmids expressing full-length IKKε, the truncated IKKε variant, or TBK1 as explained in Figure 4. In all the experiments, empty pcDNA3.1 vector plasmid was added to obtain an equal amount of DNA per well. Lipofectamine 2000 (Thermo Fisher Scientific) was used as the transfection reagent according to the manufacturer’s instruction. At 16 hours after the transfection, the cells were lysed, and the luciferase activity was measured with Dual-Glo Luciferase assay system (E2920, Promega) according to the manufacturer’s instruction.

### Lentiviral transduction of primary fibroblasts.

Lentiviral vector constructs were generated by NEBuilder HiFi DNA Assembly (New England Biolabs) and as previously described (58). For lentiviral transduction, primary fibroblasts were seeded at the density of 1 × 10^5^ cells/well in 6-well plates. Then, 24 hours later, lentiviral particles expressing EGFP-tagged WT IKBKE, EGFP-tagged IKBKE variant (c.312delC), or EGFP (as a control) at MOI 12 were added together with polybrene at final concentration of 8 μg/mL in complete media. The culture media were changed after 16 hours, and cells were left resting for 24 hours before being infected with HSV-2 at MOI 1 and 3. The transduction efficiency, as assessed by EGFP expression, ranged between 80% and 90%.

### Viral yield assays.

For measurement of virus yield, we used 2 assays, viral plaque assay and median TCID_50_ assay. For viral plaque assay, Vero cells were seeded in 12-well plates in complete Iscove’s media (basal Iscove’s, 10% FBS, 1% gentamicin [G1264, MilliporeSigma], 1% l-glutamine, and 1% mercaptoethanol [4227.3, Carl Roth GmbH]) and cultured to confluent monolayers. Supernatants from experimental cultures were then added in serial dilutions. The supernatants were removed after 1 hour, and cells were covered with a mixture of 50% media (Iscove’s, 2% FBS, 1% gentamicin) and 50% methylcellulose. After 3 days in 37°C, 5% CO_2_ incubation, the media were removed, and the cells were fixed with methanol and then stained with crystal violet (C3886, MilliporeSigma). Plaques were visualized using a light microscope and counted to calculate the plaque-forming units per milliliter of supernatant collected from the infected cells. For TCID_50_ assay, supernatants collected from infected cells were titrated in an endpoint dilution assay on Vero cells as described previously for HSV (59) and measles virus (42).

### Immunostaining.

Primary fibroblasts were transduced with lentiviral particles containing EGFP as a marker and infected with HSV-2. The cells were washed with PBS and fixed with 4% paraformaldehyde in PBS for 15 minutes. After fixation, cells were permeabilized with 0.1% Triton X-100 in PBS and blocked with 3% BSA in PBS. Cells were incubated overnight with mouse monoclonal anti–HSV-2 ICP5 primary antibody (ab6508, Abcam) at 4°C, followed by 1-hour incubation with anti-mouse Alexa Fluor 568–conjugated secondary antibody (Invitrogen, A-11004) at room temperature. Hoechst 33342 (H3570, Life Technologies) was used for nuclear staining. Images were taken directly from the plates using a fluorescence microscope (EVOS FL Cell Imaging System, Thermo Fisher Scientific). Images taken in blue, green, and red channels were merged in FIJI software.

IPSC-derived microglia and neurons were washed with PBS and fixed with 4% paraformaldehyde in PBS for 20 minutes at room temperature (RT). The cells were permeabilized with 0.3% Triton X-100 in TBS for 15 minutes at RT and incubated with blocking buffer (0.3% Triton X-100 and 5% donkey serum in TBS) for 1 hour at room temperature. Primary antibodies were diluted in blocking buffer. The following primary antibodies were used for staining: IBA1 (ab5076, Abcam) and TUJ1 (ab14545, Abcam). Samples were incubated with primary antibodies overnight at 4°C. Cells were washed 3 times with TBS and incubated for 1 hour with secondary antibodies diluted 1:500 in blocking buffer. Cells were washed 3 times with TBS and counterstained using DAPI. Samples were imaged using a Nikon A1 inverted confocal microscope.

### Flow cytometry.

Primary fibroblasts were transduced with lentiviral particles containing EGFP as described above. At 72 hours postinfection, cells were washed in PBS, dissociated into single cells with TrypLE Express (12604013, Gibco), centrifuged at 350*g* for 5 minutes at RT, resuspended in the FACS flow, and passed through a 70 μm cell strainer. Fibroblasts were gated for GFP signals using FACSLyric instrument (BD Biosciences).

### Generation of human iPSC-derived microglia and cortical neurons.

iPSC-derived microglia and cortical neurons were generated as previously described (60–62). For microglia, the human iPSC line WTSIi015-A (EBiSC through MilliporeSigma) was maintained on Matrigel (Corning) in mTeSR1 medium (Stemcell Technologies). iPSC colonies were dissociated into single cells using TrypLE Express. A total of 4 × 10^6^ iPSCs were seeded per Aggrewell 800 (Stemcell Technologies) in a 24-well plate in 2 mL mTeSR1 medium supplemented with 10 μM ROCK inhibitor, 50 ng/mL BMP-4, 20 ng/mL SCF, and 50 ng/mL VEGF-121 (all from PeproTech). Cells were cultured for 4 days in Aggrewells to form embryonic bodies with half media change (1 mL) every day. Embryonic bodies were harvested using an inverted cell strainer (40 μm), and around 15 embryonic bodies were plated per 6 wells in X-VIVO 15 medium (Lonza) supplemented with 2 mM Glutamax, 100 U/mL penicillin, 100 μg/mL streptomycin, 55 μM β-mercaptoethanol, 100 ng/mL human M-CSF (PeproTech), and 25 ng/mL human IL-3 (Cell Guidance Systems). Every 7 days 2 mL media were replaced. After around 30 days, primitive macrophage precursors could be harvested during the media change and plated, at a density of 10^5^ cells/cm^2^, in contained Advanced DMEM F12 medium (Gibco) supplemented with 2 mM Glutamax, 100 U/mL penicillin, 100 μg/mL streptomycin, 55 μM β-mercaptoethanol, 100 ng/mL human IL-34 (PeproTech), and 10 ng/mL human GM-CSF (PeproTech) and differentiated for 7 days with full media change every second day.

For neurons, the iPSCs were passaged using EDTA (Thermo Fisher Scientific) and pooled 2:1. The following day, medium was switched to neural maintenance media (NMM). NMM consisted of DMEM/F12 and neurobasal media (1:1) supplemented with 1× N2 supplement, 1× B27 supplement, 50 μM 2-mercaptoethanol, 0.5× nonessential amino acids, 100 μM l-glutamine (all from Life Technologies), 2,500 U/mL penicillin/streptomycin (GE Healthcare, now Cytiva), 10 μg/mL insulin, and 0.5 mM sodium pyruvate (both from MilliporeSigma). NMM was further supplemented with 500 ng/mL mouse Noggin/CF chimera (R&D Systems) and 10 μM SB431542 (Stemgent). The cells were maintained in NMM for 10–12 days. The cells were then dissociated in colonies using 10 mg/mL Dispase II (Thermo Fisher Scientific) and seeded on laminin-coated plates (1–2 μg/cm^2^; MilliporeSigma) in NMM supplemented with 20 ng/mL FGF2 (PeproTech). The cells were kept in FGF2-supplemented medium for 4 to 5 days and then further passaged with Dispase 2 times before day 25. After 25 days, the colonies were passaged and expanded using StemPro Accutase (Thermo Fisher Scientific) until day 35. On day 35, the cells were passaged a last time onto plates coated with 1–2 μg/cm^2^ laminin at a density of 5 × 10^4^ cells/cm^2^ in NMM. The cells were then cultured for 2 weeks before they were used for experiments. The purity of cell types was estimated by staining for Iba1 (microglia) and β-III tubulin (neurons). For both cell types, more than 95% of the cells were positive for the respective marker. To neutralize type I IFN bioactivity in supernatants from microglia, neurons were cultured with Human Type 1 IFN Neutralizing Antibody Mixture (PBL) 30 minutes before addition of culture supernatants from microglia (1:100).

### RNAi knockdown in iPSC-derived microglia and neurons.

Human iPSC-derived microglia and neurons were transfected with siRNA and Lipofectamine RNAiMAX reagent (Life Technologies) according to the manufacturer’s protocol. Final siRNA concentration used was 200 nM. iPSC microglia and neurons were used for experiments on day 4 after transfection. The siRNAs used were control siRNA (Ambion Silencer Select, 4390843), *IKBKE* siRNA (Dharmacon On-target plus, L-003723-00-0005), *TBK1* siRNA (Dharmacon On-target plus, L-003788-00-0005), *TLR3* siRNA (Ambion Silencer Select, 4530), and *cGAS* siRNA (Ambion Silencer Select, 129127).

### Cell viability assay for iPSC-derived neurons.

The iPSC-derived neurons were treated with the media collected from *IKBKE* WT and knockdown microglia before and after HSV-2 infection. At 16 hpi, neuronal cell viability was tested using CyQUANT XTT kit (X12223, Invitrogen) according to manufacturer’s instruction. Neurons were incubated for 2 hours with working reagent of the kit before measuring the absorbance.

### Statistics.

Experiments were performed in experimental triplicates and repeated a minimum of 3 times unless otherwise stated. Data were analyzed using nonparametric Mann-Whitney *U* test or 2-tailed Student’s *t* test, depending on whether the data were normally distributed. Ordinary 1-way ANOVA or Brown-Forsythe and Welch’s ANOVA tests were used for data sets including multiple groups and same conditions, as described in the respective figure legends. Analysis was done with Prism version 9.3.1 (GraphPad Software). Tests were 2 sided, and *P* values of less than 0.05 were considered significant.

### Study approval.

The patients provided oral and written consent and were included in this study in accordance with the Helsinki Declaration and guidelines after approval from the Swedish Ethical Review Authority, Gothenburg, Sweden (no. 234-17, 905-12, 2020-02527).

### Data availability.

Underlying data for the manuscript can be accessed in the Supporting Data Values file.

## Author contributions

AR, THM, KE, and SRP designed the experiments; MS, who is the patients’ physician, identified, diagnosed, and cared for patients and collected patient material; AR, MKS, SF, RN, FR, MBV, AS, MBI, and MC conducted experiments and acquired data; AR, THM, KE, and SRP analyzed data; AR wrote the manuscript (first draft); and AR, THM, KE, and SRP wrote the manuscript (editing). All authors read and approved the final version of the manuscript.

## Supplementary Material

Supplemental data

Supporting data values

## Version 1 11/08/2023

Electronic publication
