# Supplementary material for: An IKBKE variant conferring functional cGAS/STING pathway deficiency and susceptibility to recurrent HSV-2 meningitis
Source: JCI Insight. 2023 Nov 8;8(21):e173066. doi: 10.1172/jci.insight.173066 (PMC10721272; doi:10.1172/jci.insight.173066)
Supplement: Supplemental data [file jciinsight-8-173066-s190.pdf]

Supplemental Tables and figures

Supplemental Table 1. Immunophenotyping of PBMCs from P1

Lymphocytes

T-helper cells (Th)

**Cell type**	**Phenotype**	**% of CD3$^+$**	**X 10^9/L**	**Reference range* X 10^9/L**	**% Of CD3$^+$4$^+$**
T cells	CD3+	100	1,80	**0,7 - 2,1 (>16 yr, adults)**	
Th	CD3+4+	71,86	1,29	**0,3 - 1,4 (>16 yr, adults)**	100,00
Naive Th	CD3+4+197+45RA+	40,69	0,73	**0,26 – 0,38**	56,62
Recent thymic emigrants (RTEs)	CD3+4+197+45RA+31+	23,95	0,43	**0,12 – 0,24**	33,33
Effector Th	CD3+4+197-45RA+	0,01	0,00	**0 – 0,02**	0,02
Central memory Th	CD3+4+197+45RA-	26,89	0,48	**0,20 – 0,34**	37,42
Effector memory Th	CD3+4+197-45RA-	3,80	0,07	**0,11 – 0,22**	5,28
CD45RA+ effector memory Th (EMRA)	CD3+4+197-45RAlow	0,39	0,01	**0 – 0**	0,54
Activated naive Th	CD3+4+197+45RA+38+DR+	0,06	0,00	**0 – 0**	0,09
Activated effector Th	CD3+4+197-45RA+38+DR+	0,00	0,00	**0 – 0**	0,00
Activated central memory Th	CD3+4+197+45RA-38+DR+	0,30	0,01	**0 – 0**	0,41
Activated effector memory Th	CD3+4+197-45RA-38+DR+	0,25	0,00	**0 – 0,01**	0,35
Activated CD45RA+ effector memory Th (EMRA)	CD3+4+197-45RAlow38+DR+	0,00	0,00	**0 – 0**	0,00
T helper cell type1 (Th1)	CD3+4+183+196-	23,95	0,43	**0,13 – 0,29**	32,57
T helper cell type 2 (Th2)	CD3+4+45R0+183-196-194+	3,85	0,07	**0,03 - 0,05**	5,23
T helper cell type 17 (Th17)	CD3+4+183-196+	2,92	0,05	**0,06 - 0,08**	3,97
T follicular helper cell (Tfh)	CD3+4+45R0+183-196-194-185+	1,99	0,04	**0,02 - 0,02**	2,70

Cytotoxic T-cells (Tcy)

**Cell type**	**Phenotype**	**% of CD3$^+$**	**X 10^9/L**	**Reference range* X 10^9/L**	**% Of CD3$^+$8$^+$**
Tcy	CD3+8+	24,62	0,44	**0,2 - 0,9 (>16 yr, adults)**	100,00
Naive Tcy	CD3+8+197+45RA+	15,48	0,28	**0,07 – 0,13**	62,88
Effector-Tcy	CD3+8+197-45RA+	0,54	0,01	**0,01 – 0,13**	2,20
Central memory Tcy	CD3+8+197+45RA-	4,85	0,09	**0,03 – 0,05**	19,70
Effector memory Tcy	CD3+8+197-45RA-	2,60	0,05	**0,06 – 0,10**	10,55
CD45RA+ effector memory Tcy (EMRA)	CD3+8+197-45RAlow	1,20	0,02	**0,04 – 0,12**	4,86
Activated naive Tcy	CD3+8+197+45RA+38+DR+	0,03	0,00	**0 – 0**	0,14
Activated effector Tcy	CD3+8+197-45RA+38+DR+	0,06	0,00	**0 - 0**	0,25
Activated central memory Tcy	CD3+8+197+45RA-38+DR+	0,10	0,00	**0 - 0**	0,39
Activated effector memory Tcy	CD3+8+197-45RA-38+DR+	0,16	0,00	**0 - 0**	0,67

Activated CD45RA+ effector memory Tcy (EMRA)	CD3+8+197-45RAlow38+DR+	0,05	0,00	**0 - 0**	0,22
Double positive T cells	CD3+4+8+	1,75	0,03		
Double negative T cells	CD3+4-8-	1,76	0,03		

Regulatory T-cells

**Cell type**	**Phenotype**	**% of CD3^{+}4^{+}**	**X 10^9/L**	**Reference range* X 10^9/L**
Naïve Treg	CD3+4+194+25^{hög}127^{låg}45RO-	3,71	0,05	**0,02 – 0,02**
Memory Treg	CD3+4+194+25^{hög}127^{låg}45RO+	4,27	0,06	**0,02 – 0,04**
Activated naive Treg	CD3+4+194+25^{hög}127^{låg}45RO-DR+	0,01	0,00	**0 - 0**
Activated memory Treg	CD3+4+194+25^{hög}127^{låg}45RO+DR+	0,94	0,01	**0,01 - 0,01**

B cells

**Cell type**	**Phenotype**	**% of CD19^{+}**	**X 10^9/L**	**Reference range* X 10^9/L**
B cells	CD19	100	0,29	**0,1 - 0,5 (>16 yr, adults)**
Naive B cells	19+20+27-24^{låg}38^{låg}IgD+IgM+	67,12	0,19	**0,08 - 0,14**
Transitional B cells	CD3-19+20+27-24^{hög}38^{hög}IgD+IgM+	8,44	0,02	**0,01 – 0,01**
Marginal zone like B cells	CD3-19+20+27+IgD+IgM+	8,94	0,03	**0,01 – 0,03**
IgM+ memory B cells	CD3-19+20+27+IgD-IgM+	4,50	0,01	**0,01 – 0,01**
swIg (swiched Ig) memory B cells	CD3-19+20+27+IgD-IgM-	5,06	0,01	**0,01 – 0,03**
Plasma blasts	CD3-19+20-38+27^{hög}	0,78	0,00	**0 - 0**
CD21 low memory B cells	CD19+20+27+21low	3,43	0,01	**0,01 - 0,01**
CD21 low naive B cells	CD19+20+27-21low	3,92	0,01	**0,01 - 0,03**

Monocytes, NK-cells and dendritic cells

**Cell type**	**Phenotype**	**% of CD14^{+}**	**X 10^9/L**	**Reference range* X 10^9/L**
Classical monocytes	CD14+16-	78,62	0,33	**0,26 – 0,38**
Non-classical monocytes	CD14+16+	21,38	0,09	**0,06 – 0,08**
		**% of CD3^{-}56^{+}**		
NK cells	CD3-56+	100	0,35	**0,09 - 0,6 (>16 yr, adults)**
CD16+ NK cells	CD3-56+16+	95,23	0,33	**0,12 – 0,20**
CD16- NK cells	CD3-56+16-	4,74	0,02	**0,02 – 0,04**
		**% of MNC**		
Dendritic cells (DC)	CD3-19-20-14-	1,32	0,04	**0,02 – 0,02**
Myeloid DC	CD3-19-20-14-11c+	0,65	0,02	**0,01 – 0,01**
Plasmacytoid DC	CD3-19-20-14-123+	0,38	0,01	**0 - 0**

Assessment: High number of CD4: naive cells, RTE, central memory cells, Treg, CD8: Naive cells, central memory cells and B Naive cells, low numbers CD4: effector memory cells, compared to adult blood donors (ref. Value for adults used)

Supplemental Table 2. Gene variants identified in P1

Gene	Region	Variant (transcript)	Variant (protein)	CADD score	ExAC Frequency	gnomAD Frequency
RNF220	Exonic	c.45C>T	p.Y15Y	10.85	0,001	0,002
IKBKE	Exonic	c.57delC; c.312delC	p.F105fs*19; p.F105fs*36; p.F20fs*19	32.00	0,002	0,001
WIPI12	Intronic; 5'UTR	c.-79A>G; c.212-113A>G; c.158-113A>G; c.-221-113A>G		< 10	0,007	0,006
ADAMTS13	Exonic; ncRNA; 3'UTR	c.251G>A; n.2530G>A; c.3602G>A; c.3863G>A; c.*1164G>A ; c.3695G>A	p.S84N; p.S1201N; p.S1288N; p.S1232N	22.60	0,007	0,008
CCND1	Exonic	c.820G>T	p.E274*	37.00	0,001	0
DHX58	Intronic; Promoter	c.2235+3G>A; c.-1706G>A		16.24	0,003	0,006
HUWE1	Exonic	c.6927T>C; c.6900T>C	p.T2300T; p.T2309T	11.55	0,001	0,001

Supplemental Table 3. Serological status for virus infections for P1 and family

Virus / individual	**P1**	**Mother**	**Son**
HSV-1	negative	positive	negative
HSV-2	positive	negative	negative
VZV	positive	positive	positive
CMV	positive	not tested	not tested
EBV	positive	not tested	not tested
Morbilli	positive	not tested	not tested
SARS-CoV-2 (spike)	positive	not tested	not tested
SARS-CoV-2 (nucleocapsid)	negative	not tested	not tested

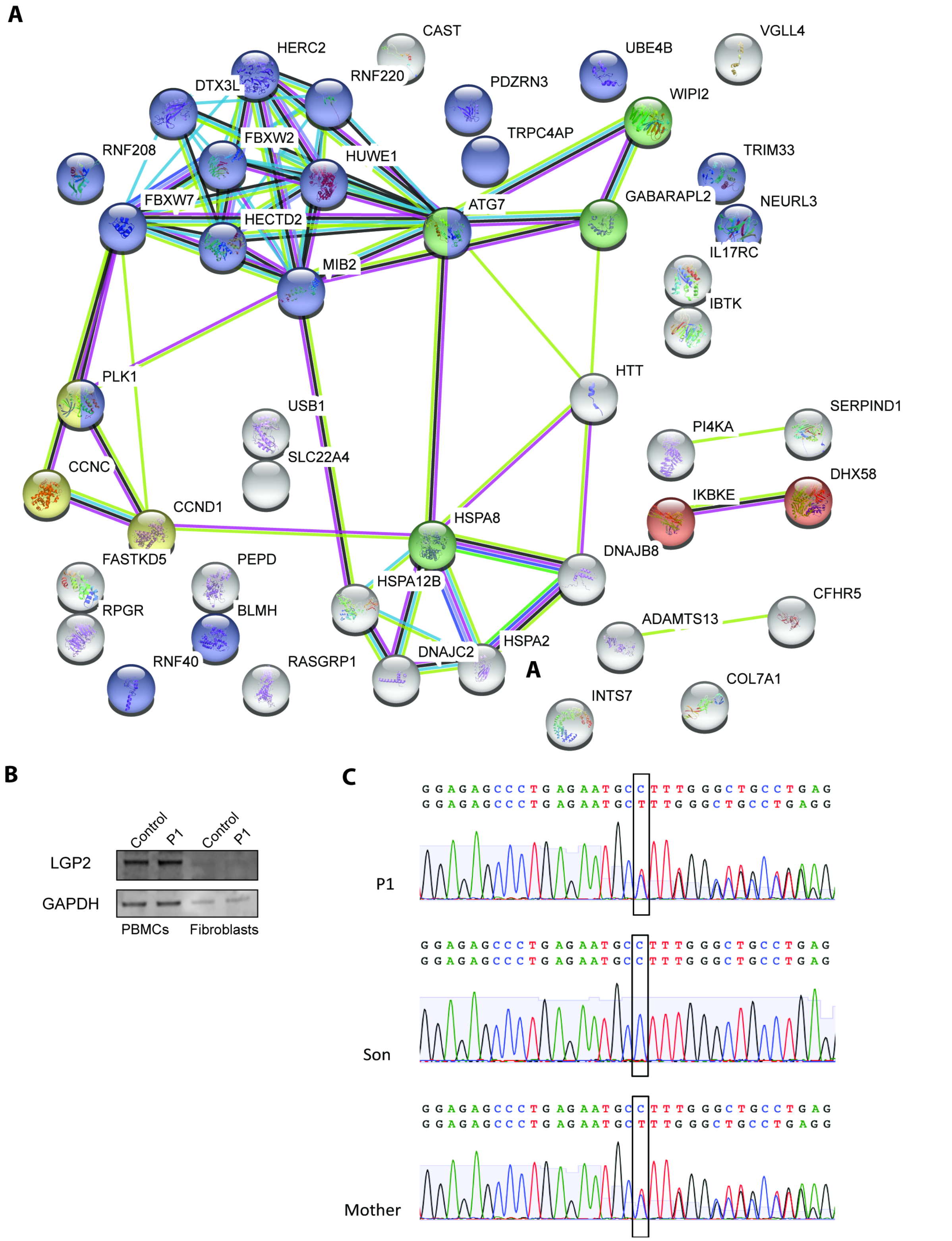

Supplemental Figure 1. Analysis of proteins encoded by identified mutant genes

(**A**) STRING protein-protein interaction network for proteins encoded by genes identified to harboring mutations in the HSV-2 meningitis cohort. Each node represents the protein affected by at least one variant in the cohort. Ubiquitin system-related proteins are indicated as blue circles, Autophagy-related proteins in green, proteins in cell cycle regulation are shown in yellow, and proteins related to type I IFN responses are in red. (**B**) Expression levels of LGP2 protein in lysates from P1 fibroblasts and PBMCs compared to controls. (**C**) Sanger sequencing around the identified mutation in IKBKE in P1, the mother, and the son.

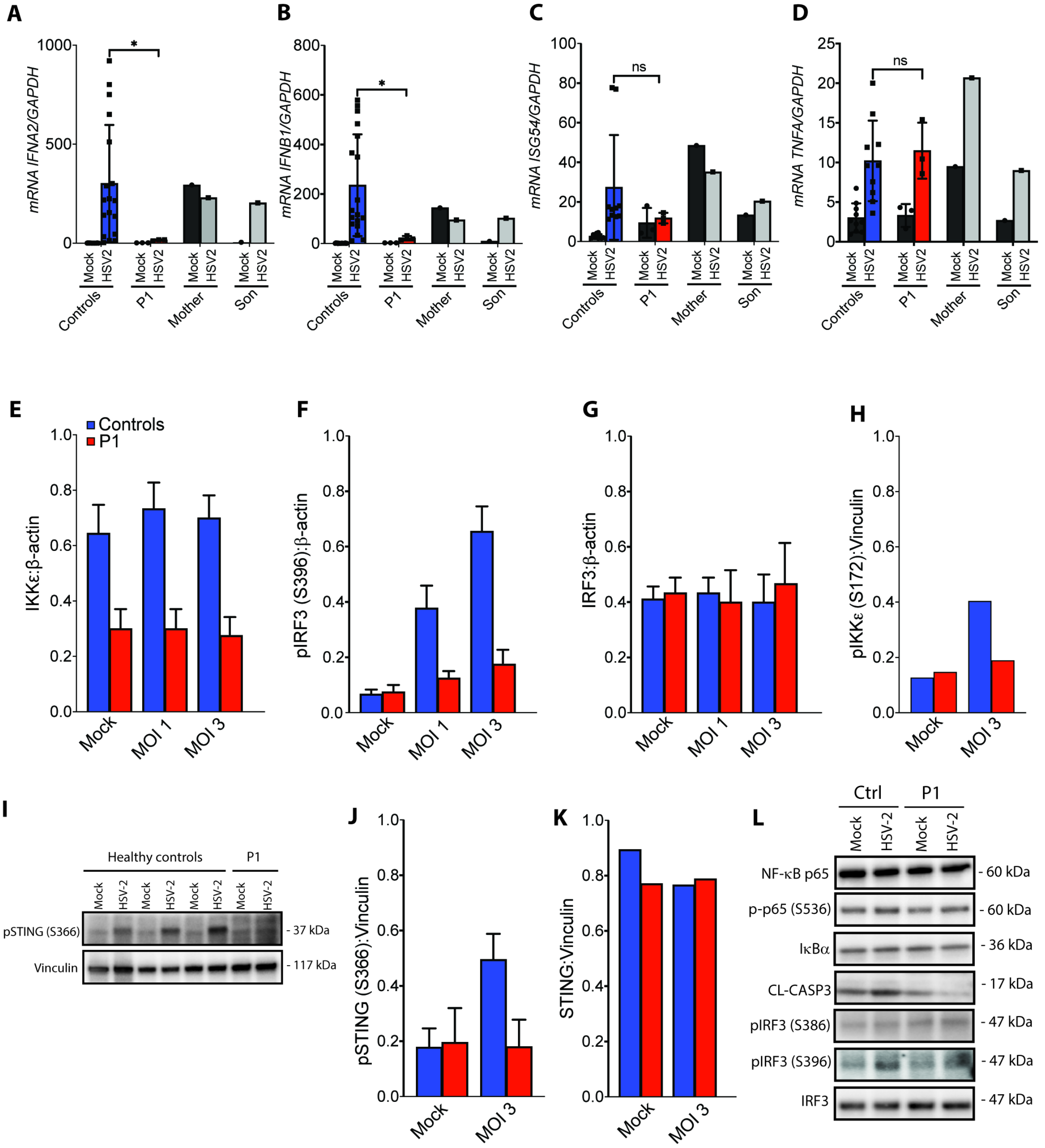

Supplemental Figure 2. Impaired IFNB1 response and STING activation in PBMC from P1
(**A-D**) PBMC from controls, the patient, her mother, and her son were infected with HSV-2 at MOI of 1. Total RNA was isolated 6 h later for measurement of the mRNA levels of *IFNA2* and *IFNB1*, the IFN-stimulated gene *ISG54*, and proinflammatory cytokine *TNFA* by RT-qPCR. All measurements were done in triplicates and normalized to *GAPDH* and further normalized to the normalized ratio of uninfected PBMC pooled from 10 healthy controls. Results were obtained from three independent experiments except for mother and son PBMC, which were examined once. The nonparametric Mann-Whitney ranked sum test was used to evaluate statistical significance between groups. Error bars representing standard error of mean (SEM). *, $P \leq 0.05$; **, $P \leq 0.01$; ***, $P \leq 0.001$; ****, $P \leq 0.0001$. (**E-H, J-K**) Graphs showing the average of signal intensity of the protein bands of patient PBMC comparing data from four WB experiments on patient lysates to 9 healthy controls. The data were quantified using ImageJ software. Error bars representing standard error of mean (SEM). (**I, L**) Whole cell lysates from PBMCs from three controls (I) or one control (L) and the patient treated with HSV-2 at MOI of 3 for 18h was examined for levels of phosphorylated STING (I), NF-kBp65, p-p65(S536), IκBα, CL-CASP3, pIRF3 (S386), pIRF3 (S396) and IRF3 (L). Vinculin protein level was used as a loading control. Data presented is from one representative experiment out of two.

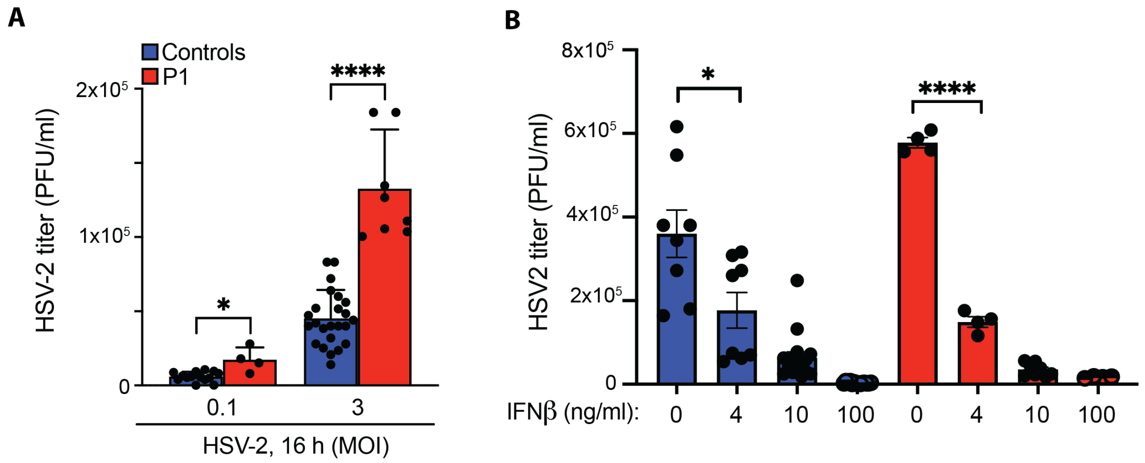

Supplemental Figure 3. Impaired IFNB1 expression and increased viral load in HSV-2-infected P1 fibroblasts

(**A**) Plaque assay was used to measure the viral load in supernatants from patient and control fibroblasts after 24 hours of HSV-2 infection at MOI of 0.1 and 3. Nonparametric Mann-Whitney rank sum test was used for statistical analysis. (**B**) Fibroblasts from P1 and from healthy controls were treated with recombinant IFNβ at the indicated doses for 6 h before infection with HSV-2 at MOI 3. Plaque assay was performed on supernatants isolated 24 h post infection. Unpaired t-test was used for statistical analysis. (A,B) Data shown is representative of two performed independent experiments. Error bars representing SEM and *, $P \leq 0.05$; **, $P \leq 0.01$; ***, $P \leq 0.001$; ****, $P \leq 0.0001$.

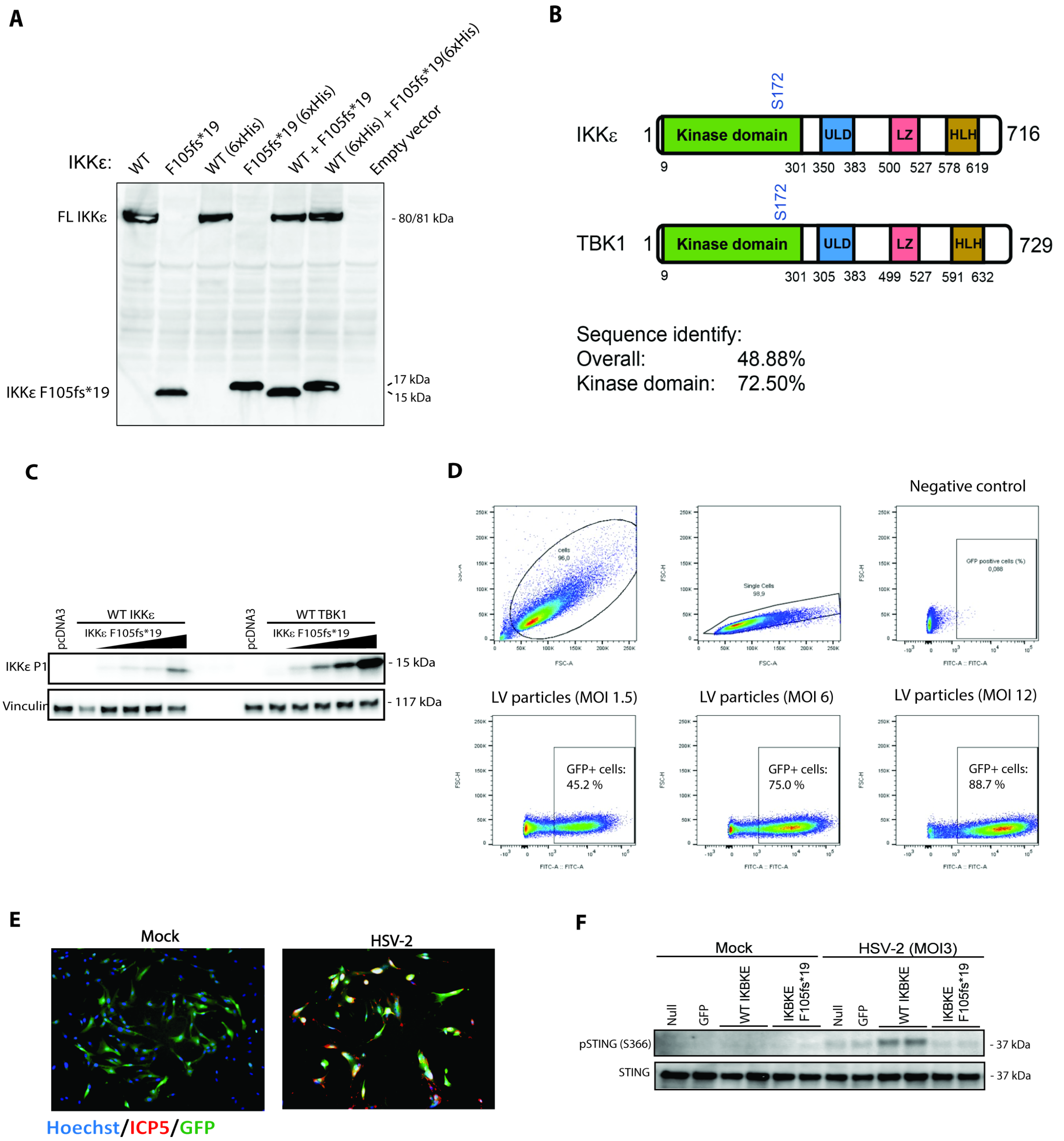

Supplemental Figure 4. The IKKε F105f*19 variant exerts dominant negative activity.
(**A**) The expression level of full-length (FL) and P1 variant IKKε in HEK293T cells was compared by transfection of equal amounts of full-length (IKBKE WT), truncated IKBKE (patient), WT-6xHis-tagged and patient-6xHis-tagged expressing plasmids, followed by Immunoblotting using an antibody recognizing the N-terminal region of IKKε. (**B**) Illustration of domain organization and sequence similarity of human IKKε and TBK1. (**C**) HEK293T cells were transfected with constructs encoding full-length (FL) TBK1 and IKKε together with increasing amounts of the *IKBKE* c.312delC variant encoding P1 IKKε. Cells were lysed after 24 h and immunoblotted for vinculin and IKKε using the antibody targeting the N-terminal epitope. (**D**) Flow cytometric analysis of fibroblasts transduced with eGFP encoding lentiviral vector shows high efficiency of transduction at MOI of 6 and 12. (**E**) Immunofluorescence images of primary fibroblast transduced with eGFP encoding lentiviral vector, which reconfirm the high transduction efficiency (green colour) in uninfected (left image) and HSV-2 infected (right image) fibroblasts. HSV-2 staining is shown in red (right image) and nuclear staining with Hoechst is in blue. (**F**) Fibroblasts from a healthy controls were transduced with no vector or lentiviruses encoding eGFP, WT IKKε, or F105fs*19. The cells were infected with HSV-2 (MOI3) for 6 h, and lysates were immunoblotted for pSTING (S366), and total STING.

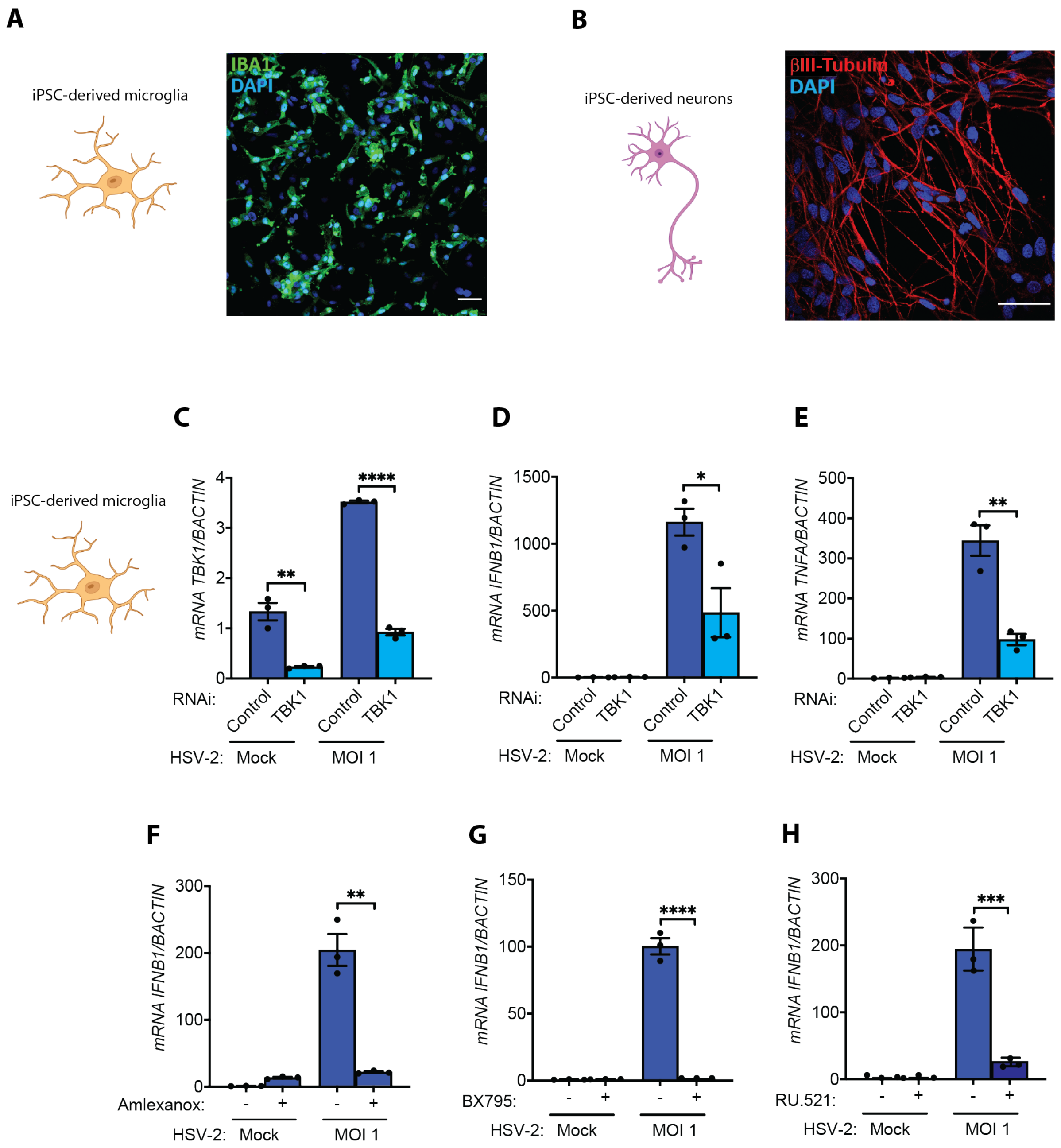

Supplemental Figure 5. Differentiation of iPSC CNS cells and response to HSV2 infection

(**A-B**) iPSCs were differentiation into (A) microglia or (B) neurons. The cells subjected to microglial differentiation were probed for the microglia marker Iba1, while the cells subjected to neuron differentiation were probed for the marker TUJ1. Cell nuclei were stained with DAPI. Scale bar, 50 μm. (**C-E**) iPSC derived microglia were transfected with *TBK1* siRNA. The efficiency of targeting was measured by RT-qPCR of *TBK1*. Microglia were infected with HSV-2 at MOI of 1 for 6 hours and mRNA level of *IFNB1 and TNFA* were analysed and normalised to *BACTIN*. **(F-G)** iPSC derived microglia were pre- and co-treated with Amlexanox (150 μg/ml), BX795 (10μM) or RU.521 (2 μg/ml) from 2 hours before HSV-2 infection at MOI of 1. mRNA level of *IFNB1* was analysed and normalised to *BACTIN*. **(C-G)** Data presented are from one (in triplicates), representative of two independent experiments performed. Unpaired t-test was used for statistical analysis. Error bars representing SEM and *, P ≤ 0.05; **, P ≤ 0.01; ***, P ≤ 0.001; ****, P ≤ 0.0001.

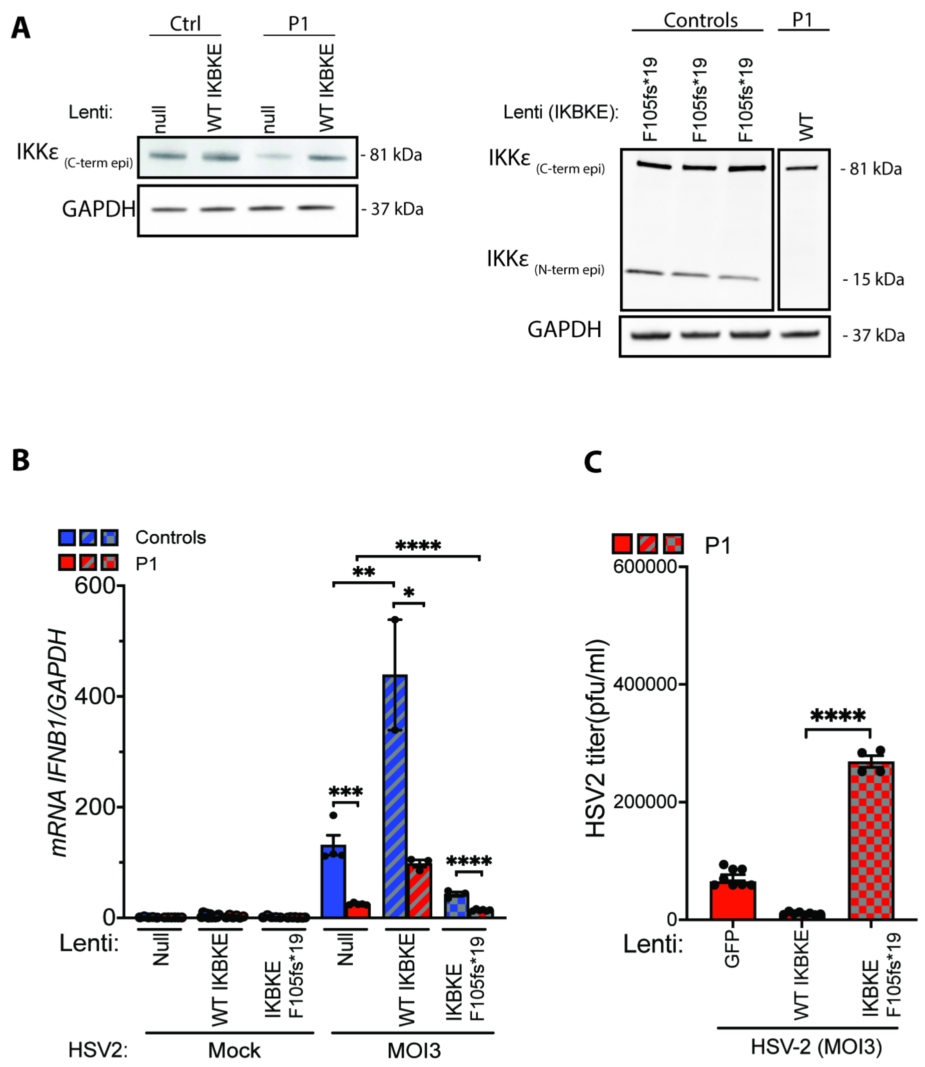

Supplemental Figure 6. Efficiency of lentiviral transduction of fibroblasts and effects on antiviral response

(A) The expression level of WT IKKε and P1 variant F105fs*19 in fibroblasts was compared by lentiviral transduction of full-length (WT IKBKE) and truncated IKBKE (IKBKE), followed by Immunoblotting using an antibody recognizing the N-terminal region of IKKε. **(B-C)** Fibroblasts from four healthy donors and P1 were transduced with lentiviral vectors encoding only GFP, full-length IKKε (WT IKBKE) and the patient variant (IKBKE F105fs*19) (MOI of 12). The cells were infected with HSV-2 at MOI 3. (B) Total RNA was harvested from cells 6 h post infection and subjected to RT-qPCR for measurement of *IFNB1* mRNA level. Data presented is from one representative of three independent experiments performed. (C) Supernatants were collected from the cells 24 h after infection for quantification of viral load by plaque assay. Data presented is merged of two independent experiments performed. Unpaired t-test was used for statistical analysis. Error bars representing SEM and *, $P \leq 0.05$; **, $P \leq 0.01$; ***, $P \leq 0.001$; ****, $P \leq 0.0001$.
